# Bridging the Gap in Breast Cancer Dormancy: Models, Mechanisms, and Translational Challenges

**DOI:** 10.3390/ph18070961

**Published:** 2025-06-26

**Authors:** Hussein Sabit, Shaimaa Abdel-Ghany, Yasser Albrahim, Al-Hassan Soliman Wadan, Sanaa Rashwan, Rebekka Arneth, Borros Arneth

**Affiliations:** 1 Department of Medical Biotechnology, College of Biotechnology, Misr University for Science and Technology, P.O. Box 77, Giza 3237101, Egypt; 2 Department of Environmental Biotechnology, College of Biotechnology, Misr University for Science and Technology, P.O. Box 77, Giza 3237101, Egypt; 3 Ministry of Health, Alahsa 39182, Saudi Arabia; 4 Oral Biology Department, Faculty of Dentistry, Galala University, Galala Plateau, Attaka, Suez 15888, Egypt; 5 University Hospital of Leicester NHS Trust, Leicester LE5 4PW, UK; 6 Department of Internal Medicine, Hospital of the Universities of Giessen and Marburg (UKGM), Justus Liebig University Giessen, Feulgenstr. 12, 35392 Giessen, Germany; 7 Institute of Laboratory Medicine and Pathobiochemistry, Molecular Diagnostics, Hospital of the Universities of Giessen and Marburg (UKGM), Philipps University Marburg, Baldingerstr. 1, 35043 Marburg, Germany; 8 Institute of Laboratory Medicine and Pathobiochemistry, Molecular Diagnostics, Hospital of the Universities of Giessen and Marburg (UKGM), Justus Liebig University Giessen, Feulgenstr. 12, 35392 Giessen, Germany

**Keywords:** BC, tumor dormancy, metastatic recurrence, microenvironment, translational research

## Abstract

Breast cancer (BC) poses a significant clinical challenge due to late metastatic recurrence, driven by dormant disseminated tumor cells (DTCs). This review emphasizes the urgency of addressing tumor dormancy to reduce metastatic relapse, a major contributor to BC mortality. DTCs evade conventional therapies and immune surveillance, reactivating unpredictably, thus necessitating targeted strategies. Current research is fragmented, with conflicting data, inadequate models, and a lack of biomarkers hindering progress. This review synthesizes these gaps and proposes actionable priorities, advocating for integrated, standardized approaches. It highlights the roles of single-cell multi-omics, spatial transcriptomics, and humanized long-term models in unraveling dormancy mechanisms. The review also emphasizes macrophage-targeted therapies, dormancy-specific trials, and biomarker validation, offering paths to clinical translation. Ultimately, this work emphasizes the urgent need for integrated multi-omics approaches, including single-cell and spatial transcriptomics, combined with advanced computational analysis. Moreover, this review critically analyzes the existing research landscape, meticulously identifying key gaps, and proposing concrete, forward-looking directions for both fundamental research and clinical translation in the challenging field of BC dormancy.

## 1. Introduction

Breast cancer remains a significant cause of mortality, largely due to the phenomenon of metastatic recurrence, often occurring years or even decades after seemingly successful primary treatment [1,2]. This late recurrence is attributed to the ability of DTCs to enter a state of dormancy, wherein they survive in a quiescent or slow-proliferating state, evading conventional therapies, only to reactivate and initiate metastatic growth at a later time [3,4]. Understanding the mechanisms that govern this dormancy-to-reactivation transition is crucial for developing effective strategies to prevent or treat metastatic disease and improve long-term outcomes for BC patients.

The study of BC dormancy is inherently challenging due to its complex nature and the limitations of current experimental models [5,6,7,8]. Traditional in vitro models often fail to fully recapitulate the intricate interactions between dormant cells and their microenvironment, while in vivo models, particularly in rodents, do not accurately reflect the extended latency periods observed in human disease. Nevertheless, recent studies have employed innovative approaches to better model dormancy [9] by utilizing advanced biomaterials that mimic the TME’s mechanical properties, thereby enhancing the understanding of dormant cell behavior and potential therapeutic strategies [10]. These biomimetic models provide valuable insights into the mechanisms governing tumor dormancy and may facilitate the development of targeted therapies to prevent relapse and improve patient outcomes. Such advancements in biomimetic modeling are crucial for unraveling the complex interplay between dormant tumor cells and their microenvironment, ultimately guiding the design of more effective therapeutic interventions [11]. Despite concerted efforts, current experimental models frequently fall short in fully capturing the intricate biology of breast cancer dormancy, particularly when attempting to translate findings to the clinical setting. Traditional in vitro systems, while valuable for initial mechanistic insights into metastatic dormancy [9], often fail to recapitulate the complex cellular heterogeneity, diverse intercellular communication, and biochemical cues inherent to the human tumor microenvironment. This limitation fundamentally restricts their capacity to model the nuanced microenvironmental regulation of dormancy [12]. Furthermore, while in vivo models allow for the study of disseminated tumor cells (DTCs) in a systemic context, many lack the capacity to faithfully mimic the exceptionally long latency periods observed in human dormancy [13]. This limitation often hinders the study of true long-term quiescent states and the specific mechanisms governing spontaneous or induced reactivation. Moreover, adequately reflecting the complex systemic influences, such as age-related changes, hormonal fluctuations, and the patient’s unique genetic and immune background, on DTC behavior and the host-niche interactions remains a formidable challenge across many established models, underscoring the ongoing need to integrate diverse model systems with genomic insights to decipher metastasis [14]. For instance, researchers have developed sophisticated 3D culture systems and biomimetic hydrogels that mimic the bone marrow and brain microenvironments, allowing for the investigation of dormancy in more physiologically relevant settings [15,16,17,18,19,20,21]. Furthermore, in vivo models, including the mouse intraductal model [22], are being refined to study the early stages of metastasis and dormancy. These advancements in modeling are essential for dissecting the mechanisms underlying dormancy.

Several key molecular and cellular mechanisms have been implicated in the regulation of BC dormancy. Epigenetic modifications, alterations in signaling pathways, and changes in cellular metabolism have all been shown to play a role. For example, histone deacetylase (HDAC) inhibitors have been shown to induce a dormancy phenotype by increasing Leukemia Inhibitory Factor Receptor (LIFR) expression [23], and the long non-coding RNA NR2F1-AS1 has been found to promote lung metastatic dormancy by regulating NR2F1 and ΔNp63 [24,25]. The role of specific proteins, such as N-cadherin [26,27], and transcription factors like FOXF2 [28] and ZFP281 [7] in maintaining or disrupting dormancy has also been highlighted. Moreover, the ECM and its mechanical properties [15,16,17,29,30], as well as the involvement of autophagy [31], have emerged as critical factors in regulating DTC fate.

The TME, particularly the interactions between dormant cells and stromal and immune cells, is increasingly recognized as a crucial determinant of dormancy. Natural killer (NK) cells [5] and T cells [32,33] have been shown to play a role in controlling dormancy, while hepatic stellate cells can suppress NK cell-mediated dormancy [6]. Myeloid-derived suppressor cells (MDSCs) [34] and neutrophils [25] also contribute to the complex interplay between the immune system and dormant cancer cells. Furthermore, mesenchymal stem cells (MSCs) and their secreted exosomes have been implicated in promoting dormancy [35,36,37]. The metabolic state of dormant cells and their interaction with the niche, including the role of lipid metabolism [38] and glycolysis [39], are also areas of active investigation.

Despite significant advances in our understanding of the biology of BC dormancy, several challenges remain in translating these findings into clinical practice. A major obstacle is the lack of specific biomarkers to identify dormant cells and predict their reactivation potential [40,41]. Additionally, dormant cells are inherently resistant to conventional therapies, posing a significant challenge for eradication. Emerging therapeutic strategies, such as targeting specific signaling pathways [1] or metabolic vulnerabilities [42], are being explored, but clinical translation requires further investigation. The identification of novel non-coding RNAs, such as ELEANOR [43], also presents potential therapeutic avenues. This review critically evaluates current research on BC dormancy, focusing on models, molecular and cellular mechanisms, and clinical translation challenges, while also delving into these challenges and discussing potential future research and clinical development directions.

## 2. Molecular and Cellular Mechanisms of Dormancy

BC dormancy is a complex process involving a variety of molecular and cellular mechanisms that allow DTCs to survive in a quiescent or slow-proliferating state. Understanding these mechanisms is crucial for developing effective strategies to prevent metastatic recurrence. This section will delve into the intricate molecular and cellular processes that govern dormancy, highlighting key signaling pathways, epigenetic modifications, metabolic adaptations, and the critical role of the TME.

### 2.1. Cell-Intrinsic Mechanisms: Molecular Pathways and Key Regulators

**Figure 1:**
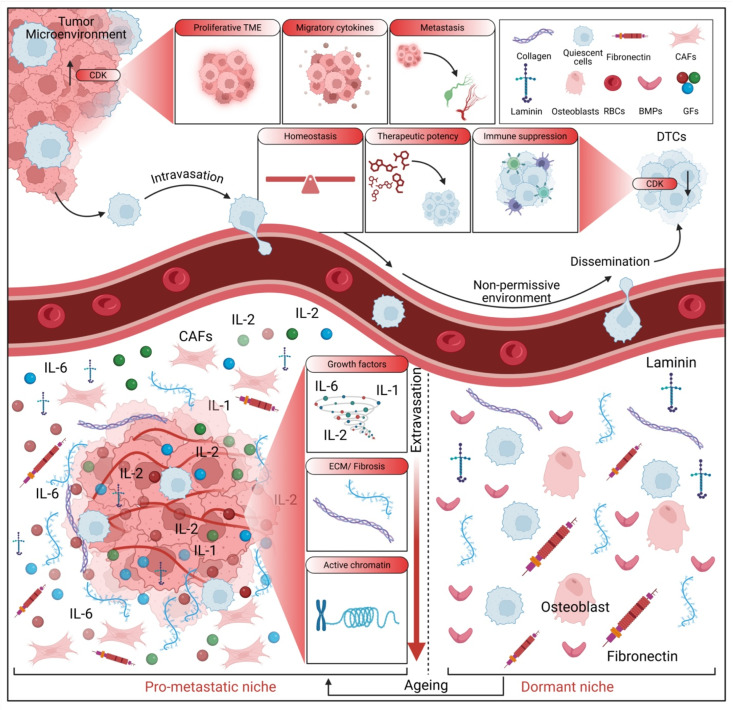
A comprehensive schematic representation of the dynamic process by which disseminated tumor cells (DTCs) transition from a proliferative TME to a dormant niche, highlighting the key stages of intravasation, dissemination, extravasation, and niche adaptation. The figure begins in the top left corner with the tumor microenvironment, where cancer cells undergo cell cycle progression driven by cyclin-dependent kinase (CDK) activity, resulting in a proliferative TME. The TME is enriched with migratory cytokines, facilitating epithelial-mesenchymal transition (EMT), invasion, and eventual metastasis. CAFs, red blood cells (RBCs), quiescent cells, bone morphogenetic proteins (BMPs), growth factors (GFs), fibronectin, collagen, laminin, and osteoblasts are illustrated as critical components within the TME. Tumor cells then intravasate into the bloodstream, becoming circulating tumor cells (CTCs), which can disseminate to distant organs. Once in circulation, DTCs encounter a non-permissive environment that impedes immediate colonization. Some DTCs retain active CDK signaling, while others undergo immune suppression and therapeutic resistance. As DTCs reach secondary sites, they may extravasate into local tissue, where microenvironmental factors determine their fate. The bottom left quadrant shows the establishment of a pro-metastatic niche characterized by an abundance of CAFs and elevated levels of interleukins IL-1, IL-2, and IL-6, as indicated by arrows marking their local secretion. This niche supports DTC proliferation and is rich in ECM components such as fibronectin and laminin, as well as an active chromatin structure that allows for transcriptional activity, promoting tumor growth and colonization. The bottom right quadrant contrasts this with a dormant niche, where the microenvironment favors DTC quiescence. Here, ECM components like laminin and fibronectin, along with interactions with osteoblasts, contribute to cellular dormancy. The chromatin in these cells is likely inactive or condensed, and signaling pathways are less conducive to proliferation. The entire process is modulated over time by aging, as shown by the downward arrow from the pro-metastatic niche to the dormant niche, suggesting that age-related changes in the ECM, cytokine levels, and stromal composition contribute to the shift from a proliferative to a dormant state. Created by BioRender (https://www.biorender.com).

Several cell-intrinsic mechanisms play a crucial role in the initiation and maintenance of dormancy (Figure 1). These mechanisms involve a complex interplay of signaling pathways, transcriptional regulators, and post-translational modifications that ultimately lead to a state of cellular quiescence and survival. Signaling pathways involved in dormancy include the PI3K/AKT/mTOR pathway, which is frequently dysregulated in cancer and often downregulated in dormancy, leading to decreased protein synthesis and cell cycle arrest. Studies have shown that inhibition of mTOR can induce a dormancy-like state in various cancer cell lines [44,45], and its modulation is critical for the transition to and maintenance of a quiescent state. TGF-β signaling is another important pathway; TGF-β is a multifunctional cytokine with both tumor-suppressing and tumor-promoting effects. While typically acting as a tumor suppressor in early-stage cancer, inducing cell cycle arrest and apoptosis, it can promote invasion and metastasis in later stages [46,47,48,49]. During dormancy, TGF-β signaling can play a complex role, potentially both inducing and maintaining quiescence, and some studies suggest that TGF-β can activate p38 mitogen-activated protein kinases (MAPK), a kinase involved in cell cycle arrest and stress responses, thereby promoting dormancy [50,51,52,53]. MAPK signaling, involving MAPKs such as ERK, JNK, and p38, also plays a role in various cellular processes, including proliferation, differentiation, and apoptosis [54,55]. The role of MAPKs in dormancy is context-dependent, with p38 MAPK activation being associated with cell cycle arrest and dormancy [53]. Transcriptional regulation is also crucial, with specific transcription factors playing a critical role in regulating the expression of genes involved in cell cycle arrest, survival, and metabolism, thereby promoting dormancy. For instance, the FOX family of transcription factors has been implicated in the regulation of quiescence [28].

ZFP281 is another transcription factor involved in maintaining or disrupting dormancy [7]. The long non-coding RNA NR2F1-AS1 has also been found to promote lung metastatic dormancy by regulating NR2F1 and ΔNp63 [24,56]. Cell cycle regulators, such as cyclin-dependent kinases (CDKs) and their inhibitors (CDKIs), are also key, with the expression of CDKIs like p21 and p27 often upregulated during dormancy, leading to cell cycle arrest at the G0/G1 phase, a hallmark of dormant cells that allows them to survive for extended periods without proliferating [57].

This complex interplay of cell cycle regulation and apoptosis evasion highlights the multifaceted strategies dormant cells employ to maintain their quiescent state and resist therapeutic interventions [58]. Finally, apoptosis inhibitors are essential for dormant cell survival, as these cells must evade apoptosis for prolonged periods, and the expression of anti-apoptotic proteins, such as Bcl-2 and Mcl-1, is often increased in dormant cells, protecting them from cell death [1,5,59].

### 2.2. Epigenetic Modifications: Shaping the Dormancy Landscape

Beyond the genetic code, epigenetic modifications stand as critical arbiters of gene expression and, ultimately, cellular identity. These dynamic alterations, particularly DNA methylation and histone modifications, profoundly influence a DTC’s capacity to both enter and steadfastly maintain a dormant state [60]. A fundamental understanding of tumor dormancy is essential, as this state permits cancer cells to remain viable yet non-proliferative, often eluding detection and conventional treatment. Consequently, a precise dissection of how intricate epigenetic changes contribute to this perplexing phenomenon is critical for advancing effective cancer therapies [61].

One of the foundational mechanisms of epigenetic regulation lies in histone modification. These modifications, whether they involve acetylation, methylation, or other alterations, possess the remarkable ability to either activate or repress gene expression, depending entirely on their specific context and precise genomic location [62,63,64,65,66]. Consider histone acetylation and methylation: these are not static marks but rather highly dynamic processes that actively remodel chromatin structure, thereby governing gene accessibility. Histone deacetylases (HDACs), for instance, function by removing acetyl groups from histones, which leads to a more condensed chromatin structure, effectively silencing gene transcription. Intriguingly, studies have demonstrated that inhibiting these HDACs can induce a dormancy phenotype, notably by increasing LIFR expression [23]. This finding strongly suggests that controlling histone deacetylation is a pivotal mechanism in promoting the quiescent state essential for dormancy. For instance, epigenetic silencing of BMP6 by the SIN3A-HDAC1/2 repressor complex has been linked to melanoma metastasis, highlighting the broad impact of HDAC activity [67]. Conversely, the precise location and context of histone methylation can dictate whether it activates or represses gene expression, adding another layer of regulatory complexity.

Complementing histone modifications, DNA methylation stands as another critical epigenetic mechanism profoundly influencing gene expression. The addition of a methyl group to cytosine residues within DNA, particularly in promoter regions, typically results in transcriptional repression, effectively “switching off” genes that might otherwise be necessary for tumor growth [68]. Interestingly, dormant cells exhibit distinctly altered DNA methylation patterns, strongly implying that this mechanism contributes to their unique, altered gene expression profiles [69]. Conversely, a decrease in DNA methylation (*aka* hypomethylation) can enhance gene transcription, a shift that may contribute to the reactivation of dormant tumors under certain conditions.

These fascinating insights into the dynamic interplay between DNA methylation and tumor dormancy underscore the immense potential for targeted epigenetic therapies, aiming to disrupt this quiescent state and prevent devastating recurrence. Recent long-term multimodal recording studies have shed further light on the epigenetic adaptation routes in dormant BC cells, revealing how these cells dynamically adjust their epigenetic landscape to persist in a quiescent state [60,70,71,72]. Furthermore, research is actively revealing the role of epigenetic modifiers and DNA oxidation in the cell-autonomous regulation of cancer stem cells, which often exhibit dormant properties [73]. The complex interplay between histone modifications and DNA methylation adds yet another layer of fascinating complexity to the precise regulation of gene expression within tumor cells. As dormant cells inherently possess unique epigenetic landscapes, unraveling these intricate modifications promises to provide profound insights into the fundamental mechanisms governing tumor behavior and, critically, their potential for unpredictable reactivation [71,74]. These findings collectively underscore the profound impact of epigenetic mechanisms in shaping the dormancy landscape, offering promising avenues for therapeutic interventions aimed at either maintaining dormancy or inducing reactivation for subsequent eradication.

### 2.3. Metabolic Adaptations: Surviving in a Quiescent State

**Figure 2:**
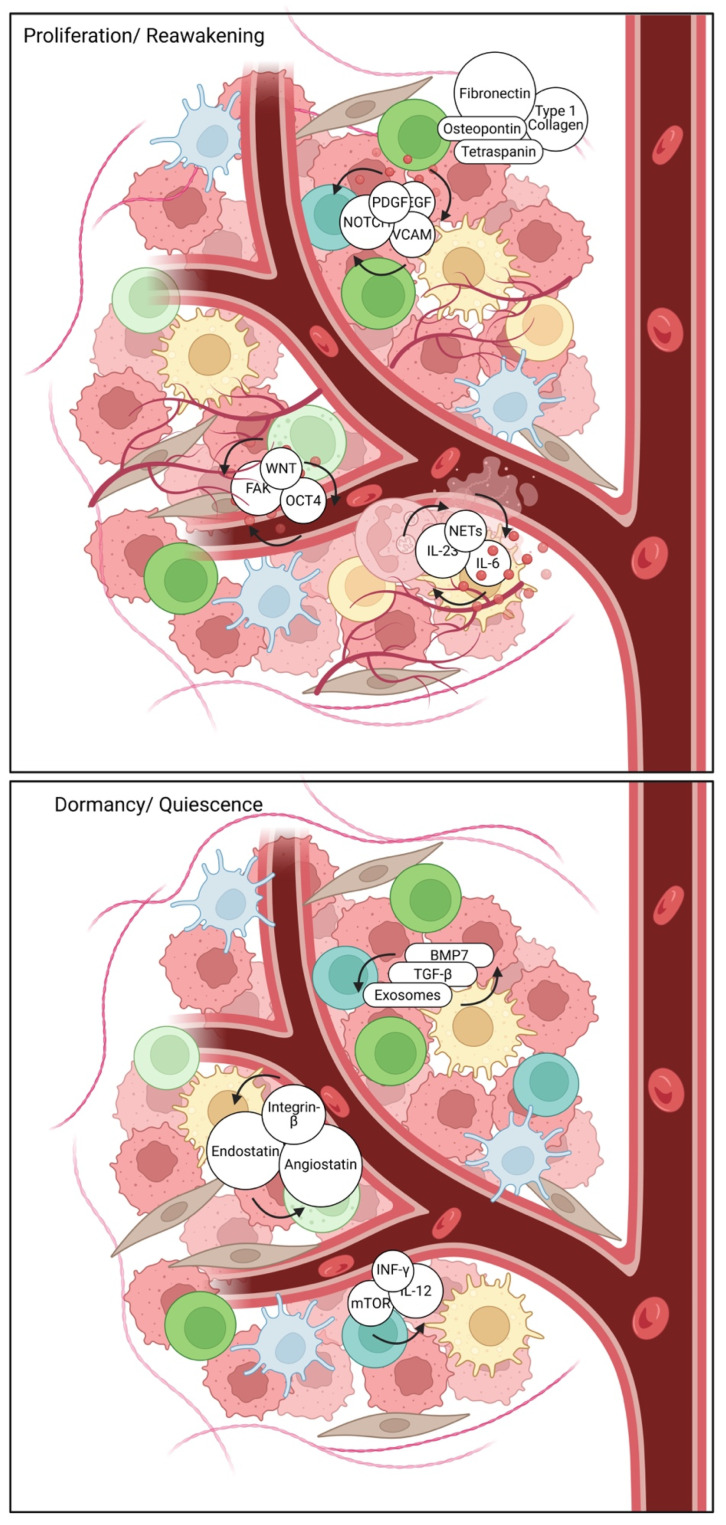
Dynamic modulation of the TME governing cancer cell dormancy versus proliferation. This two-panel schematic illustrates the distinct molecular and cellular landscapes that dictate either the reawakening/proliferation or dormancy/quiescence of cancer cells within the metastatic niche. In the upper panel (“Proliferation/Reawakening”), the TME supports metastatic outgrowth through active interactions between cancer cells, stromal components, and immune cells. Pro-growth factors including PDGF, FGF, WNT, VCAM, and NOTCH, along with transcriptional regulators like OCT4 and FAK, are secreted by stromal and endothelial cells, while fibroblasts and extracellular matrix (ECM) components such as fibronectin, type I collagen, osteopontin, and tetraspanins facilitate cell adhesion, survival, and invasion. Inflammatory cytokines such as IL-6 and IL-23, together with neutrophil extracellular traps (NETs), further stimulate cancer proliferation and immune evasion. Conversely, the lower panel (“Dormancy/Quiescence”) portrays a suppressive TME enriched with dormancy-inducing signals. Bone morphogenetic protein 7 (BMP7), TGF-β, and exosomes derived from stromal cells, in concert with immune-regulatory cytokines like IL-12 and IFN-γ, contribute to a non-proliferative state. Anti-angiogenic factors including angiostatin, endostatin, and integrin-β help prevent neovascularization, maintaining cancer cells in a latent, non-dividing condition. This balance between proliferative signaling and dormancy cues is tightly controlled by the spatial and temporal orchestration of CAFs, immune infiltrates, and ECM remodeling, with CAFs emerging as key modulators. They can either promote reawakening through the secretion of proliferative cytokines and matrix proteins or support dormancy via TGF-β and exosomal communication, making them central targets in preventing cancer recurrence and metastasis. Created by BioRender.

The remarkable ability of dormant cancer cells to persist for extended periods, often years or even decades, hinges critically on their sophisticated metabolic adaptations (Figure 2). These cellular rewiring events allow them to not only survive in a quiescent, non-proliferative state but also to withstand harsh microenvironmental cues and evade therapeutic interventions. At a fundamental level, dormant cells typically exhibit a significantly reduced overall metabolic activity compared to their actively proliferating counterparts [75]. This metabolic downregulation is a crucial energy-conserving strategy, minimizing energy expenditure and, importantly, reducing the production of reactive oxygen species (ROS), which can otherwise cause detrimental damage to cellular components and macromolecules [76]. The precise calibration of this metabolic quiescence is vital; too much activity risks detection, while too little could lead to cellular death.

A key metabolic shift observed in many dormant cell populations involves a move away from glycolysis, traditionally considered the primary energy-producing pathway in rapidly proliferating cancer cells, towards a greater reliance on oxidative phosphorylation (OXPHOS). This shift, while seemingly counterintuitive for cancer cells, is fundamentally more efficient in terms of ATP yield per glucose molecule, albeit at a slower rate. This metabolic reorientation helps conserve scarce glucose resources within the hostile dormant niche and reduces the accumulation of lactate, a byproduct of glycolysis that can acidify the microenvironment [39]. However, it is important to acknowledge that the metabolic landscape of dormant cells is not uniform.

Some investigations have indeed indicated a continued or even specific role for glycolysis in certain dormant states, suggesting nuanced and context-dependent metabolic plasticity [77]. This inherent adaptability allows dormant cells to respond to varying nutrient availability and microenvironmental signals. For instance, cells in hypoxic niches might still rely more heavily on anaerobic glycolysis even in a dormant state, albeit at a reduced rate, highlighting the dynamic equilibrium influenced by their immediate surroundings [78]. The precise interplay between glycolysis and OXPHOS in supporting dormancy remains an active area of investigation.

Beyond glucose metabolism, lipid metabolism emerges as a profoundly crucial component in supporting the quiescent state, providing both an alternative energy source and essential building blocks for maintaining membrane integrity under duress. Indeed, disrupted lipid metabolism and the involvement of apolipoproteins have been directly implicated in the intricate mechanisms governing tumor dormancy [38,79]. Dormant cells frequently enhance their reliance on fatty acid oxidation (FAO) to generate ATP, utilizing stored lipids to fuel their reduced energetic demands [80]. This metabolic flexibility allows them to leverage a more sustained energy supply, crucial for long-term survival in nutrient-deprived environments. Recent studies have further detailed how specific lipid droplet accumulation contributes to a more quiescent phenotype and protects against oxidative stress in dormant cells [81,82,83,84]. These lipid droplets not only serve as energy reserves but also act as protective reservoirs for lipophilic molecules, buffering against cellular damage. This suggests that targeting lipid metabolic pathways, perhaps by inhibiting FAO or disrupting lipid droplet formation, might represent a novel therapeutic approach to disrupt dormancy and sensitize these cells to subsequent treatments [85].

Moreover, autophagy, a fundamental cellular process involving the regulated degradation and recycling of damaged organelles and proteins, has emerged as a critical factor in regulating DTC fate [31]. Autophagy serves as an essential mechanism for maintaining cellular homeostasis, particularly under stressful conditions like nutrient deprivation or therapeutic insult. Importantly, dormant cells often exhibit a significant upregulation of autophagy, allowing them to survive under nutrient-limiting conditions, recycle cellular components, and efficiently remove damaged structures that might otherwise trigger apoptosis or senescence [86,87]. This sustained autophagic activity not only fuels survival but also likely plays a role in reducing the metabolic activity that might expose dormant cells to detection or therapeutic vulnerability [88]. For example, by breaking down long-lived proteins and dysfunctional mitochondria, autophagy ensures that the cellular machinery remains efficient and minimal, reducing the energetic burden [89]. The extent to which autophagy contributes to therapeutic resistance in dormant cells, and whether its judicious modulation could “reawaken” these cells or enhance their susceptibility to treatment, is a compelling and clinically relevant area of ongoing research. Understanding these intricate metabolic adaptations provides a critical foundation for designing innovative therapeutic strategies aimed at selectively targeting and eliminating these elusive dormant cell populations, ultimately improving long-term outcomes for cancer patients.

### 2.4. The TME: A Critical Regulator of Dormancy

The TME emerges as a profoundly influential regulator of DTC dormancy. Indeed, this intricate milieu is a complex network, comprising stromal cells, immune cells, ECM components, and an array of soluble factors, all capable of either fostering or suppressing dormancy [90,91]. The ECM, for instance, is far more than mere structural scaffolding; it critically governs cell adhesion, migration, and survival. Its composition and mechanical properties are particularly influential in determining the ultimate fate of DTCs [15,16,17,29,30]. Intriguingly, changes in ECM stiffness, composition, and organization can profoundly alter critical signaling pathways within DTCs, tipping the delicate balance towards either quiescence or reactivation. A compelling example is astrocytic laminin-211, which has been shown to actively drive disseminated breast tumor cell dormancy specifically within the brain microenvironment [92]. This intricate interplay between ECM stiffness and cellular signaling pathways underscores the critical importance of precisely targeting the TME for effective therapeutic intervention.

Beyond the ECM, various stromal cells, including fibroblasts, endothelial cells, and pericytes, are instrumental in sculpting the TME. These cells exert significant influence over DTC dormancy through their secretion of growth factors, cytokines, and ECM components [93]. Notably, MSCs and their secreted exosomes have also been implicated in promoting dormancy [35,36,37]. Gaining a deeper understanding of the precise mechanisms by which these diverse stromal cells interact with DTCs is paramount, as it can provide invaluable insights into potential therapeutic strategies aimed at preventing metastatic relapse. Targeting the TME, particularly the intricate communications between stromal cells and DTCs, thus holds considerable promise for enhancing therapeutic efficacy and substantially reducing the risk of metastatic recurrence in BC patients.

The immune system, a fascinatingly complex entity, demonstrates a dual role in regulating dormancy. While natural killer (NK) cells [5] and T cells [32,33] are known to exert control over dormancy, it is equally important to acknowledge that certain components, like hepatic stellate cells, can suppress NK cell-mediated dormancy [6]. This dynamic interplay between NK cells and hepatic stellate cells, for instance, is critically important in determining the ultimate fate of DTCs; indeed, even subtle alterations in their interactions can significantly impact metastatic outcomes. Furthermore, myeloid-derived suppressor cells (MDSCs) and neutrophils [25] also contribute to this complex immunologic interplay within the TME. The soluble factors abundant in the TME, including a wide array of growth factors, cytokines, and chemokines, can also significantly influence DTC dormancy. For example, TGF-β has been shown to play a role in both inducing and maintaining quiescence. Ultimately, a comprehensive understanding of these multifaceted interactions within the TME is essential for developing targeted therapies aimed at preventing the unpredictable reactivation of dormant DTCs and, consequently, improving patient outcomes [94]. This multifaceted approach underscores the critical need for comprehensive strategies that address both the immune and stromal components of the TME to effectively manage cancer dormancy and recurrence.

However, while the TME is a pivotal factor in regulating DTC dormancy, it is crucial to recognize that dormancy itself is a multifactorial phenomenon influenced by numerous biological processes extending beyond the TME. To assert that the TME, with its stromal cells, immune cells, and ECM, is the sole primary regulator of DTC fate might oversimplify the inherent complexity of cancer biology [95]. While the ECM certainly provides structural support and can influence cellular behaviors, the intrinsic properties of DTCs, including their specific genetic and epigenetic makeup, also play a critical role in determining their dormancy and subsequent reactivation potential [96]. Moreover, the mechanical properties of the ECM and its precise composition may not always be as decisively influential as sometimes suggested. DTCs often exhibit remarkable plasticity and adaptability to diverse microenvironments, indicating that their fate can be profoundly influenced by their intrinsic signaling pathways and genetic alterations, sometimes even more so than by external factors like ECM stiffness. While the role of stromal cells is undoubtedly significant, it should not overshadow the equally critical importance of intrinsic tumor cell characteristics that can dictate dormancy somewhat independently of the surrounding TME.

### 2.5. The Role of Autophagy

Autophagy is fundamental catabolic process involving the degradation and recycling of cellular components, plays a nuanced yet critical role in sustaining cancer cell dormancy. In the quiescent state, dormant disseminated tumor cells (DTCs) often leverage basal or stress-induced autophagy to maintain metabolic homeostasis, survive various microenvironmental adversities, including nutrient deprivation, hypoxia, and therapeutic stress, and prevent their own death [97,98]. This cellular recycling mechanism provides essential building blocks and energy, enabling DTCs to endure prolonged periods without proliferation, effectively contributing to their long-term survival and intrinsic resistance to conventional therapies [99]. Crucially, autophagy has been identified as a prerequisite for mammary tumor recurrence, facilitating the survival of dormant tumor cells following therapy [86]. Moreover, the re-epithelialization of cancer cells, a process relevant to dormancy and relapse, can increase autophagy and DNA damage, further implicating its role in this complex state [31].

Modulating autophagy thus presents intriguing therapeutic opportunities in breast cancer [100,101]. On the one hand, autophagy inhibition is being actively explored as a strategy to disrupt the survival mechanisms of dormant cells, making them more vulnerable to metabolic stress or subsequent therapeutic interventions. By blocking this essential recycling pathway, dormant cells may accumulate toxic waste products or deplete critical energy reserves, potentially forcing them out of quiescence or inducing cell death [102]. For instance, targeting PIK3C3-mTORC1 signaling, which regulates autophagy, in dormancy-prone breast cancer cells can blunt metastasis initiation [1]. Conversely, in certain contexts, autophagy activation might also be considered to push cells towards autophagic cell death or sensitize them to other pro-apoptotic stimuli, highlighting its multifaceted functions [103]. A significant challenge in targeting autophagy lies precisely in its context-dependent nature: it can act as both a pro-survival and a pro-death mechanism, making selective disruption difficult without unintended consequences. Therefore, precise timing and sophisticated combination strategies are essential to selectively disrupt dormancy without inadvertently promoting tumor progression. Future research focusing on the specific autophagic pathways active in dormant breast cancer cells could lead to more targeted and effective therapeutic interventions.

### 2.6. Therapeutic Implications and Challenges

A thorough comprehension of the molecular and cellular mechanisms underpinning dormancy holds substantial implications for the development of novel therapeutic strategies to prevent or treat metastatic disease. Targeting these mechanisms offers the potential to either eradicate dormant cells or effectively impede their reactivation. Specifically, interventions aimed at crucial signaling pathways involved in dormancy, such as the PI3K/AKT/mTOR pathway or various MAPK pathways, could disrupt the quiescent state of these cells and promote cell death [104].

Beyond signaling pathways, epigenetic modifying agents, including HDAC inhibitors or DNA methyltransferase inhibitors, represent a promising avenue to alter the epigenetic landscape of dormant cells, thereby increasing their susceptibility to therapeutic interventions [105]. Similarly, exploiting the distinctive metabolic adaptations of dormant cells, such as their reliance on specific metabolic pathways, could facilitate their selective elimination [106]. Furthermore, immunotherapeutic approaches, which encompass stimulating anti-tumor immune responses or abrogating immunosuppressive mechanisms, could be employed to target and eliminate dormant cell populations. The concept of dormant tumor cell vaccination, for instance, has been explored as a potential strategy [107].

It is crucial to consider that, while a thorough understanding of the molecular and cellular mechanisms of dormancy is undeniably vital, a singular focus on targeting these mechanisms for therapeutic purposes may simplify the complexities of cancer biology [108,109]. The proposition that dormant cells can be simply eradicated or prevented from reactivating through targeted therapies while appealing, may be an oversimplification. For instance, disrupting crucial signaling pathways such as PI3K/AKT/mTOR or MAPK could, inadvertently, induce compensatory mechanisms within the cancer cells, potentially leading to more aggressive tumor behavior rather than promoting cell death [110]. Furthermore, the universal efficacy of epigenetic modifying agents, including HDAC inhibitors or DNA methyltransferase inhibitors, cannot be assumed due to the heterogeneous nature of dormant cells, which may exhibit varied responses to such treatments depending on their unique genetic and epigenetic contexts.

**Figure 3:**
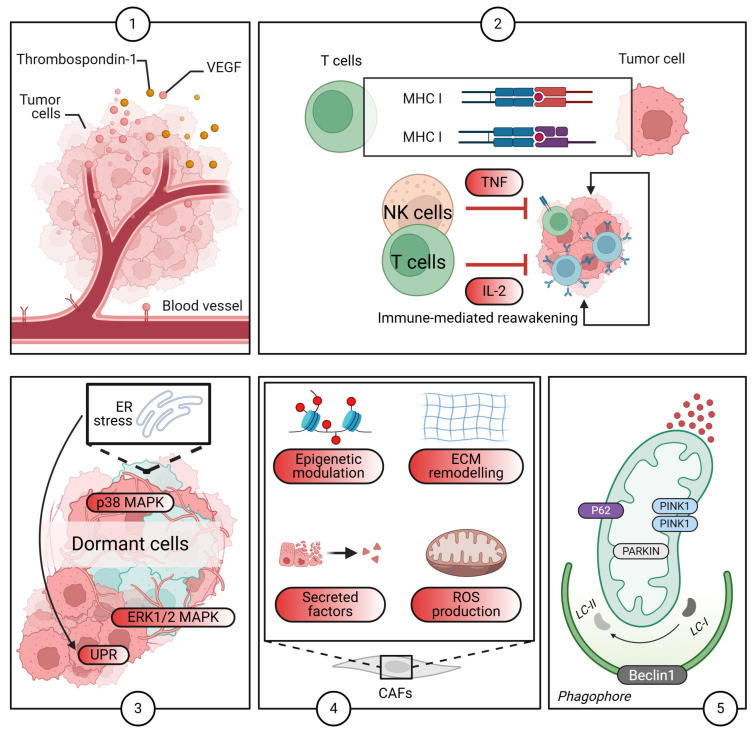
Molecular and microenvironmental pathways regulating breast cancer dormancy. This figure illustrates the major interconnected mechanisms underlying the maintenance and reactivation of dormant breast cancer cells. (1) The angiogenic switch is tightly regulated by pro- and anti-angiogenic factors; Thrombospondin-1 (TSP-1) inhibits angiogenesis and supports dormancy, while VEGF (vascular endothelial growth factor) promotes neovascularization and enables outgrowth of dormant tumor cells near blood vessels. (2) Immune surveillance plays a dual role—T cells and NK cells can either sustain dormancy or mediate reawakening through cytokines such as IL-2 and TNF; the expression of MHC-I on tumor cells modulates recognition and immune editing. (3) Cellular stress responses such as the unfolded protein response (UPR) are activated by ER stress and influence dormancy via the balance between p38 MAPK and ERK1/2 MAPK pathways; a low ERK\:p38 ratio favors entry into a dormant state. (4) The tumor microenvironment (TME), especially cancer-associated fibroblasts (CAFs), contributes to dormancy or reactivation via secreted factors, epigenetic remodeling, ECM alterations, reactive oxygen species (ROS) production, and metabolic reprogramming. (5) Autophagy and metabolic adaptation, particularly mitophagy mediated by the PINK1/PARKIN axis and autophagy-related components like LC3 and Beclin1, support cell survival under nutrient deprivation and stress, enabling long-term tumor cell quiescence. These interconnected pathways collectively define the dormancy landscape and represent targets for therapeutic intervention to prevent metastatic relapse. Created by BioRender.

Therefore, the establishment and maintenance of BC dormancy are governed by a multitude of complex molecular and cellular mechanisms (Figure 3). These involve both cell-intrinsic factors and complex interactions with the microenvironment, alongside nuanced metabolic adaptations. Extensive further research is unequivocally required to fully elucidate these complex processes and to identify genuinely effective therapeutic targets.

### 2.7. Current Immunotherapies and Dormant Cancer Cells: Challenges and Strategies

Current immunotherapies, particularly immune checkpoint inhibitors (ICIs), primarily rely on activating robust, pre-existing anti-tumor T cell responses to eradicate proliferating cancer cells. However, their efficacy against dormant cancer cells (DTCs) remains a significant challenge in preventing metastatic relapse, largely due to the unique characteristics of these quiescent cells. Dormant cells exhibit low immunogenicity and metabolic quiescence, characterized by minimal proliferative activity. This often translates to reduced expression of tumor antigens and downregulation of MHC-I molecules [3,111,112], rendering them “immunologically cold” and consequently difficult targets for immune recognition [113]. Furthermore, dormancy niches frequently suffer from immune exclusion and suppression, lacking significant effector T cell infiltration. DTCs may reside in immune-privileged or profoundly immunosuppressive microenvironments, devoid of the activated T cells that ICIs aim to unleash [111]. This immune-hostile landscape is often shaped by factors that contribute to cancer immune evasion dynamics. Beyond these extrinsic factors, dormant cells may possess intrinsic resistance mechanisms, employing unique survival strategies or expressing alternative immune evasion molecules not directly targeted by current ICI strategies [114].

Despite these formidable barriers, several promising strategies aim to render dormant cells susceptible to immunotherapy. A fundamental prerequisite for ICI efficacy is reactivation: if dormant cells spontaneously re-enter the cell cycle, they may regain their immunogenicity and become more visible to the immune system. This concept underpins the search for therapies that can actively “unfreeze” dormancy. The most promising approach involves combination therapies, integrating ICIs with agents that actively disrupt dormancy, enhance immunogenicity, remodel the immunosuppressive TME, or specifically target dormancy-specific survival pathways. For instance, combining ICIs with radiation therapy can induce distinct immune response trajectories in triple-negative breast cancer, potentially making dormant cells more responsive [115]. Similarly, epigenetic modulators, such as Jumonji family histone demethylase inhibitors [116] or BET bromodomain inhibitors [117], can sensitize resistant cells, offering avenues to disrupt intrinsic dormancy programs. The crucial role of autophagy in mammary tumor recurrence, by promoting dormant tumor cell survival following therapy [86], also highlights a potential vulnerability for combinatorial targeting. Ultimately, targeting dormancy-specific vulnerabilities directly, such as the dependence on PIK3C3-mTORC1 signaling in dormancy-prone breast cancer cells [1] could lead to either cell death or re-sensitization to conventional immune attack. Understanding the complex interplay of genetic factors, including those influencing HLA disruption [112] or RNA dynamics [118], in response to immunotherapy will be pivotal in overcoming dormant cell resistance and achieving durable responses.

## 3. The Metastatic Niche and Microenvironmental Control

The process of metastasis, wherein cancer cells disseminate from the primary tumor to colonize distant organs, is critically influenced by the formation of a specialized microenvironment known as the metastatic niche. This niche, orchestrated by a complex interplay of cellular and molecular components, provides a permissive environment that supports the survival, proliferation, and eventual outgrowth of metastatic cells. Recent investigations have significantly advanced our understanding of the intricate mechanisms governing metastatic niche formation and the multifaceted role of the microenvironment in controlling this process.

### 3.1. Cellular and Molecular Contributors to Metastatic Niche Formation

Beyond the established players, various cell types and molecular mediators actively contribute to the formation and remodeling of the metastatic niche. Neutrophils, for instance, have been shown to contribute to the metastatic process through several mechanisms. Cathepsin C promotes BC lung metastasis by modulating neutrophil infiltration and neutrophil extracellular trap (NET) formation [119], and aged neutrophils form mitochondria-dependent vital NETs to promote BC lung metastasis [120]. Furthermore, chronic stress has been linked to increased metastasis via neutrophil-mediated changes to the microenvironment [121], inducing pulmonary epithelial cells to produce acetylcholine that remodels the lung pre-metastatic niche of BC by enhancing NETosis [122]. Conversely, a synthetic metastatic niche model has revealed that anti-tumor neutrophils can drive BC metastatic dormancy in the lungs [123]. Moreover, lung mesenchymal stromal cells, influenced by Th2 cytokines, mobilize neutrophils and facilitate metastasis by producing complement C3 [124]. These findings collectively underscore the complex and context-dependent roles of neutrophils in modulating the metastatic microenvironment.

Macrophages are another crucial component in shaping the metastatic niche. TREM2 macrophages regulate the metastatic boundary in BC lung metastases [125], and single-cell landscapes have elucidated intratumoral heterogeneity and the immunosuppressive microenvironment in liver and brain metastases of BC [126]. Tumor-derived exosomes can drive immunosuppressive macrophages in a pre-metastatic niche through glycolytic dominant metabolic reprogramming [127], and macrophages further promote pre-metastatic niche formation of BC through aryl hydrocarbon receptor activity [128]. Prune-1, for example, drives polarization of tumor-associated macrophages (TAMs) within the lung metastatic niche in TNBC [129], and specific metastasis-associated myeloid subpopulations, including macrophages, have been identified in BC lung metastasis [130]. These studies illustrate the diverse functions of macrophages in establishing an immunosuppressive microenvironment that facilitates metastatic colonization.

**Figure 4:**
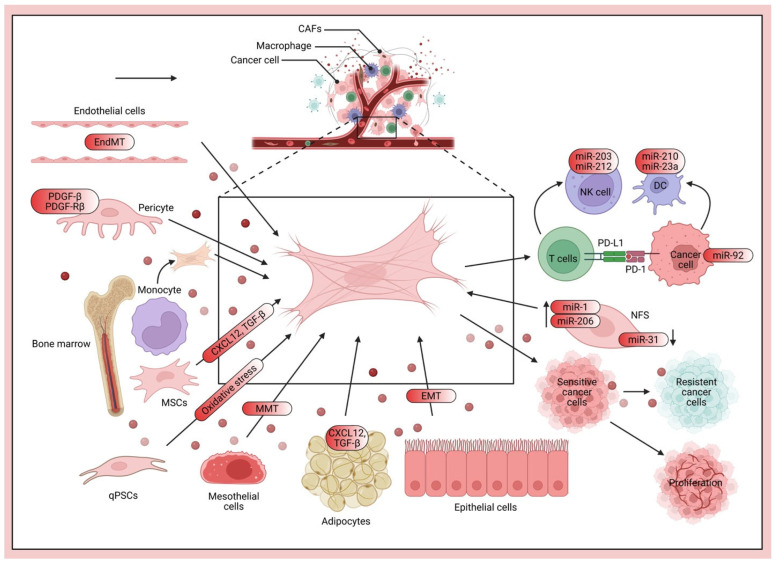
Mechanisms of CAF activation, recruitment, and contribution to tumor progression and immune evasion. This schematic illustrates the dynamic TME and the complex interplay of cellular and molecular events that lead to the activation of CAFs and their contribution to cancer growth and progression. Various cell types including endothelial cells, pericytes, monocytes, MSCs, quiescent pancreatic stellate cells (qPSCs), mesothelial cells, adipocytes, and epithelial cells can be recruited and reprogrammed into CAFs through diverse signaling pathways such as PDGF-β/PDGFR-β (pericytes), EndMT (endothelial-mesenchymal transition), EMT (epithelial-mesenchymal transition), and MMT (mesothelial-mesenchymal transition), often driven by the tumor-secreted factors including CXCL12, TGF-β, and oxidative stress. CAFs localize around tumor nests and blood vessels, interacting with cancer cells, macrophages, and immune cells to modulate tumor behavior. They contribute to immune evasion by regulating immune checkpoints such as the PD-1/PD-L1 axis and suppressing T cell activation, while also promoting an immunosuppressive environment through secretion of exosomal miRNAs such as miR-92, miR-203, miR-212 (targeting NK cells), and miR-210, miR-23a (targeting dendritic cells). These exosomal miRNAs further impair anti-tumor immunity and promote tumor immune escape. In parallel, CAFs enhance cancer cell proliferation and resistance through the secretion of soluble factors and extracellular vesicles that modulate non-sensitive fibroblasts (NFS), converting them into tumor-promoting phenotypes via miR-31, and by regulating miR-1 and miR-206 to sustain survival and adaptation of resistant cancer cells. Overall, CAFs orchestrate a multifaceted network that fuels tumor cell proliferation, enhances stemness, drives therapy resistance, and reshapes the immune microenvironment, establishing themselves as central mediators of cancer progression and critical targets for anti-cancer therapy. Created by BioRender.

Fibroblasts, key constituents of the TME, are also deeply implicated in metastatic niche formation (Figure 4).

Lung fibroblasts, for instance, facilitate pre-metastatic niche formation by remodeling the local immune microenvironment [131]. Fibroblast-derived IL33 has been shown to facilitate BC metastasis by modifying the immune microenvironment and driving type 2 immunity [132]. Furthermore, tryptophan 2,3-dioxygenase-positive matrix fibroblasts can fuel BC lung metastasis via kynurenine-mediated ferroptosis resistance of metastatic cells and T-cell dysfunction [133]. Cancer-associated fibroblasts further facilitate premetastatic niche formation through lncRNA SNHG5-mediated angiogenesis and vascular permeability in BC [134].

Exosomes, small extracellular vesicles secreted by tumor cells, have emerged as important mediators of intercellular communication in the metastatic process. Tumor-derived Cav-1 has been shown to promote pre-metastatic niche formation and lung metastasis in BC [123], and BC exosomes contribute to pre-metastatic niche formation and promote bone metastasis of tumor cells [135]. Interestingly, IRF5 has been found to suppress metastasis through the regulation of tumor-derived extracellular vesicles and pre-metastatic niche formation [136]. Moreover, extracellular vesicles secreted by TNBC stem cells trigger premetastatic niche remodeling and metastatic growth in the lungs [137]. It is also important to note that extracellular vesicle-mediated purinergic signaling contributes to host microenvironment plasticity and metastasis in TNBC [138]. TNBC-derived EVs further promote a hepatic premetastatic niche via a cascade of microenvironment remodeling [139]. CD44-positive extracellular vesicles derived from highly metastatic mouse mammary carcinoma cells play a role in pre-metastatic niche formation [140]. The incorporation of acetylated LAP-TGF-β1 proteins into exosomes has been found to promote TNBC cell dissemination in lung micro-metastasis [141]. Chronic stress-induced and tumor-derived SP1+ exosomes polarize IL-1β+ neutrophils and increase lung metastasis of BC [142]. Finally, extracellular vesicle-packaged CDH11 and ITGA5 induce the premetastatic niche for bone colonization of BC cells [143].

The bone microenvironment, a frequent site of BC metastasis, has been extensively characterized. Research indicates that the bone microenvironment increases the phenotypic plasticity of ER+ BC cells [144], and lymphotoxin-β promotes BC bone metastasis colonization and osteolytic outgrowth [145]. Furthermore, CST6 protein and peptides inhibit BC bone metastasis by suppressing CTSB activity and osteoclastogenesis [146]. RSPO2 and RANKL have been shown to signal through LGR4 to regulate osteoclastic premetastatic niche formation and bone metastasis [147]. Mammary tumor cells notably remodel the bone marrow vascular microenvironment to support metastasis [148]. Bone metastasis initiation is coupled with bone remodeling through osteogenic differentiation of NG2+ cells [149]. Engineered models of metastatic colonization of human bone marrow reveal BC cell remodeling of the hematopoietic niche [150]. Insights into the metastatic bone marrow niche have also been gained from fibronectin and β1 integrin transgenic mice [151]. Interestingly, bone marrow niche chemoprotection of metastatic solid tumors is mediated by CYP3A4 [152]. The metastatic niche in BC bone metastasis has been unraveled through single-cell RNA sequencing [153]. The early bone metastatic niche has been engineered through human vascularized immuno-bone minitissues [154]. Moreover, bone marrow NG2+/Nestin+ mesenchymal stem cells have been found to drive DTC dormancy via TGFβ2 [7].

### 3.2. Emerging Concepts and Methodological Advances in Niche Research

Additional factors and mechanisms influencing the metastatic niche continue to be identified, providing a more comprehensive view. Notably, chronic psychological stress has been shown to promote BC pre-metastatic niche formation by mobilizing splenic MDSCs via TAM/CXCL1 signaling [155]. Furthermore, a high-fat diet and platelet activation have been demonstrated to impact pre-metastatic niche formation [156]. The tumor-intrinsic NLRP3-HSP70-TLR4 axis, interestingly, drives premetastatic niche development and hyperprogression during anti-PD-1 immunotherapy [157]. Cancer-associated adipocytes (CAAs) also promote BC cell survival and metastasis [158]. The characterization of the temporal progression of lung immune remodeling during BC metastasis has been achieved [159,160,161]. It is important to note that metabolic shifts in lipid utilization and reciprocal interactions within the lung metastatic niche of TNBC have been revealed by spatial multi-omics [159]. Dietary approaches targeting metabolic adaptations in the BC-liver metastatic niche can improve endocrine therapy efficacy [162]. Furthermore, p38α stress-activated protein kinase drives the formation of the pre-metastatic niche in the lungs [163]. A sponge-like nano-system has been shown to suppress tumor recurrence and metastasis by restraining myeloid-derived suppressor cell-mediated immunosuppression and formation of the pre-metastatic niche [164]. Finally, engineered niches are increasingly being utilized to delineate the metastatic potential of BC [165,166].

Other studies have explored the role of specific signaling molecules and cellular interactions within the metastatic niche. For instance, immunotherapeutic IL-6R and targeting the MCT-1/IL-6/CXCL7/PD-L1 circuit have been found to prevent relapse and metastasis of TNBC [167]. IP-10 (CXCL10) can trigger the emergence of dormant BC cells in a metastatic liver microenvironment [168], and cystine/glutamate antiporter xCT deficiency reduces metastasis without impairing immune system function in BC mouse models [169]. Syndecan-1 and -4 have been shown to influence Wnt signaling and cell migration in human BCs [170]. Furthermore, a spatially resolved atlas of BC has revealed the intercellular machinery of the venular niche governing lymphocyte extravasation [171]. Microenvironment-tailored micelles effectively restrain carcinoma-astrocyte crosstalk for brain metastasis [172], and proteomic insights into metastatic BC response to brain cell-secreted factors have also been provided [173]. Interestingly, Brd7 loss has been found to reawaken dormant metastasis-initiating cells in the lung by forging an immunosuppressive niche [174].

Additional investigations have leveraged advanced techniques and model systems to further elucidate the complexities of the metastatic niche. Versatile ginsenoside Rg3 liposomes, for example, inhibit tumor metastasis by capturing CTCs and disrupting metastatic niches [175]. Osteoclast-derived apoptotic bodies inhibit naive CD8+ T cell activation via Siglec15, promoting BC secondary metastasis [176]. A microphysiological early metastatic niche-on-a-chip has revealed how heterotypic cell interactions and inhibition of integrin subunit β3 impact BC cell extravasation [177], and morphological phenotyping of organotropic brain- and bone-seeking mTNBC cells has also been performed [178]. Furthermore, a 3D vascularized BC model has been utilized to study the role of osteoblasts in the formation of a pre-metastatic niche [179], and thyroid status has been found to regulate the TME, delineating BC fate [180]. Computed tomography has revealed microenvironment changes in the premetastatic lung [181], and micellar nanoparticles inhibit BC and pulmonary metastasis by modulating the recruitment and depletion of myeloid-derived suppressor cells [182]. Enzymatically transformable polymer-based nanotherapeutics have been developed to eliminate minimal relapsable cancer [183]. S100A9-targeted cowpea mosaic virus has been explored as a prophylactic and therapeutic immunotherapy against metastatic BC and melanoma [184]. Gut colonization with an obesity-associated enteropathogenic microbe modulates the premetastatic niches to promote BC, lung, and liver metastasis [185], and localized degradation of neutrophil extracellular traps by photo-regulated enzyme delivery is effective for cancer immunotherapy and metastasis suppression [186]. Stromal senescence following treatment with the CDK4/6 inhibitor Palbociclib alters the lung metastatic niche and increases metastasis of drug-resistant mammary cancer cells [187], and tumor-secreted GRP78 has been found to promote the establishment of a pre-metastatic niche in the liver microenvironment [188]. Finally, stress-induced metastatic niches in BC have been discussed [189], the role of germline variants in the metastasis of breast carcinomas has been explored [190], and LSD1 deficiency in BC cells has been found to promote the formation of pre-metastatic niches [191].

### 3.3. Challenges in Targeting the Metastatic Niche

Despite significant advancements in our understanding of the molecular and cellular mechanisms of dormancy, several considerable challenges remain. One major impediment is the inherent heterogeneity of dormant cells, which often exhibit diverse phenotypes and variable responses to therapeutic interventions based on their distinct anatomical location and local microenvironment [95]. Compounding this challenge is the current paucity of specific biomarkers capable of reliably identifying dormant cells and accurately predicting their reactivation potential [192]. Developing effective strategies to overcome these limitations is paramount for enhancing patient outcomes and minimizing the risk of metastasis.

The intricate interplay between the TME and dormant cells introduces an additional layer of complexity in the development of effective therapeutic strategies. The TME actively influences the behavior of dormant cells via various signaling pathways, including those mediated by immune cells and ECM components, which can paradoxically support quiescence or facilitate subsequent reactivation [193]. For example, factors secreted by immunosuppressive cells can reinforce dormancy while concurrently establishing an environment conducive to later awakening, thereby complicating the therapeutic landscape [194,195,196]. This inherent dual role of the TME necessitates a more nuanced therapeutic approach—one that not only directly targets dormant cells but also judiciously modifies the surrounding environment to prevent their reactivation, consequently augmenting the efficacy of treatment regimens designed to combat metastatic disease.

## 4. Experimental Models: Strengths and Limitations

The fundamental study of cancer dormancy and metastasis relies critically on the judicious development and utilization of appropriate experimental models. These sophisticated models are meticulously designed to recapitulate the intricate biological processes inherent in these phenomena, thereby enabling researchers to rigorously investigate underlying mechanisms and evaluate potential therapeutic interventions. Current modeling approaches encompass a diverse range of methodologies, each possessing distinct advantages and inherent limitations.

### 4.1. In Vitro Models: 3D Cultures, Organoids, and Specialized Niches

In vitro models, particularly three-dimensional (3D) cultures and organoids, have emerged as invaluable tools for advancing our understanding of cancer dormancy. Traditional two-dimensional (2D) cell cultures often fail to adequately mimic the complex TME, thereby limiting their capacity to accurately represent in vivo conditions. In stark contrast, 3D cultures offer a more physiologically relevant environment, significantly enhancing cell-cell and cell-matrix interactions. For example, the utility of 3D models in capturing specific aspects of dormancy has been demonstrated through the use of a hyaluronic acid hydrogel to model mass dormancy in brain metastatic BC spheroids [18]. Furthermore, engineered 3D matrices have been shown to induce dormancy and reveal mechanosensitive signaling axes [10]. Bioinspired synthetic matrices have also been effectively employed for long-term 3D culture to investigate estrogen receptor-positive (ER+) BC dormancy [19], showcasing their utility for extended studies. Organoids, representing even more complex 3D structures that closely mimic the architecture and function of whole organs, are increasingly being leveraged for their enhanced physiological relevance.

Within this subsection, it is pertinent to discuss how these in vitro models are being utilized to dissect complex interactions within specialized niches, particularly concerning extracellular vesicles and specific organ microenvironments.

#### 4.1.1. Extracellular Vesicles and Niche Modeling

Extracellular vesicles (EVs), notably exosomes secreted by tumor cells, have been identified as crucial mediators of intercellular communication in the metastatic process within in vitro and ex vivo models. Investigations have shown that tumor-derived Cav-1 promotes pre-metastatic niche formation and lung metastasis in BC [123], while BC exosomes contribute to pre-metastatic niche formation and foster bone metastasis of tumor cells [135]. Interestingly, IRF5 has been observed to suppress metastasis through its regulatory role on tumor-derived EVs and pre-metastatic niche formation [136]. Moreover, EVs secreted by TNBC stem cells are capable of triggering premetastatic niche remodeling and subsequent metastatic growth in the lungs [137]. It is important to note that EV-mediated purinergic signaling contributes significantly to host microenvironment plasticity and metastasis in TNBC [138]. Furthermore, TNBC-derived EVs promote a hepatic premetastatic niche through a cascade of microenvironment remodeling events [139]. CD44-positive EVs originating from highly metastatic mouse mammary carcinoma cells also play a discernible role in pre-metastatic niche formation [140]. The incorporation of acetylated LAP-TGF-β1 proteins into exosomes has been identified as a factor promoting TNBC cell dissemination in lung micro-metastasis [197]. Additionally, chronic stress-induced and tumor-derived SP1+ exosomes, which polarize IL-1β+ neutrophils, contribute to increased lung metastasis of BC [142]. Finally, EVs packaged with CDH11 and ITGA5 have been shown to induce the premetastatic niche, facilitating bone colonization by BC cells [143].

#### 4.1.2. Modeling Organ-Specific Metastatic Niches

The bone microenvironment, a frequent and critical site of BC metastasis, has been extensively characterized using in vitro and ex vivo approaches. Research indicates that the bone microenvironment enhances the phenotypic plasticity of ER+ BC cells [144], and lymphotoxin-β promotes BC bone metastasis colonization and osteolytic outgrowth [145]. Furthermore, CST6 protein and peptides inhibit BC bone metastasis by suppressing CTSB activity and osteoclastogenesis [146]. Signaling through LGR4 by RSPO2 and RANKL has been implicated in regulating osteoclastic premetastatic niche formation and bone metastasis [147]. Notably, mammary tumor cells actively remodel the bone marrow vascular microenvironment to support metastasis [148]. Bone metastasis initiation is intricately coupled with bone remodeling via the osteogenic differentiation of NG2+ cells [149]. Engineered models of metastatic human bone marrow colonization reveal BC cell-induced remodeling of the hematopoietic niche [150]. Further insights into the metastatic bone marrow niche have been gained from studies utilizing fibronectin and β1 integrin transgenic mice [151]. Interestingly, bone marrow niche chemoprotection of metastatic solid tumors appears to be mediated by CYP3A4 [152]. The metastatic niche in BC bone metastasis has been further elucidated through single-cell RNA sequencing [153]. The early bone metastatic niche has also been engineered through human vascularized immuno-bone minitissues [154]. Moreover, bone marrow NG2+/Nestin+ mesenchymal stem cells have been identified as drivers of DTC dormancy via TGFβ2 [198].

Additional factors and mechanisms influencing the metastatic niche have been identified. Chronic psychological stress, for instance, has been shown to promote BC pre-metastatic niche formation by mobilizing splenic MDSCs via TAM/CXCL1 signaling [155]. Furthermore, a high-fat diet and platelet activation have been demonstrated to impact pre-metastatic niche formation [156]. The tumor-intrinsic NLRP3-HSP70-TLR4 axis, interestingly, drives premetastatic niche development and hyperprogression during anti-PD-1 immunotherapy [157]. Cancer-associated adipocytes also promote BC cell survival and metastasis [158]. The temporal progression of lung immune remodeling during BC metastasis has been meticulously characterized [159,160,161]. Significantly, metabolic shifts in lipid utilization and reciprocal interactions within the lung metastatic niche of TNBC have been revealed by spatial multi-omics [159]. Dietary approaches targeting metabolic adaptations in the BC-liver metastatic niche can improve endocrine therapy efficacy [162]. Furthermore, p38α stress-activated protein kinase drives the formation of the pre-metastatic niche in the lungs [163]. A sponge-like nano-system has been developed to suppress tumor recurrence and metastasis by restraining myeloid-derived suppressor cell-mediated immunosuppression and inhibiting pre-metastatic niche formation [164]. Finally, engineered niches are increasingly utilized to delineate the metastatic potential of BC [165,166].

### 4.2. In Vivo Models: Mouse Models for Systemic Insights

In vivo models, particularly various mouse models, have proven indispensable in cancer research, including the rigorous study of dormancy and metastasis. These models offer the distinct advantage of enabling the investigation of complex tumor-host interactions within a living organism, thereby providing crucial insights into systemic factors that influence disease progression. For instance, HER2/neu transgenic mice were successfully employed to demonstrate that a nano-encapsulated oral formulation of fenretinide can promote local and metastatic BC dormancy [199]. A preclinical mouse intraductal model was specifically utilized to investigate metastatic dormancy in estrogen receptor-positive BC [22]. Numerous studies have also leveraged mouse models to explore the intricate role of TME, such as the profound influence of osteoblast-derived signals on disseminated BC cells [200]. Furthermore, transcriptome analysis in mouse models has significantly contributed to our understanding of the inherent heterogeneity of metastatic BC [201]. However, it remains critical to acknowledge the inherent limitations of mouse models, particularly regarding physiological and genetic differences compared to humans. An in vivo xenograft model has been described for studying tumor dormancy, highlighting its value while implicitly recognizing the intrinsic differences between human and mouse biological systems [202].

### 4.3. Emerging Systems: Microfluidics and Advanced Methodologies for Niche Studies

**Figure 5:**
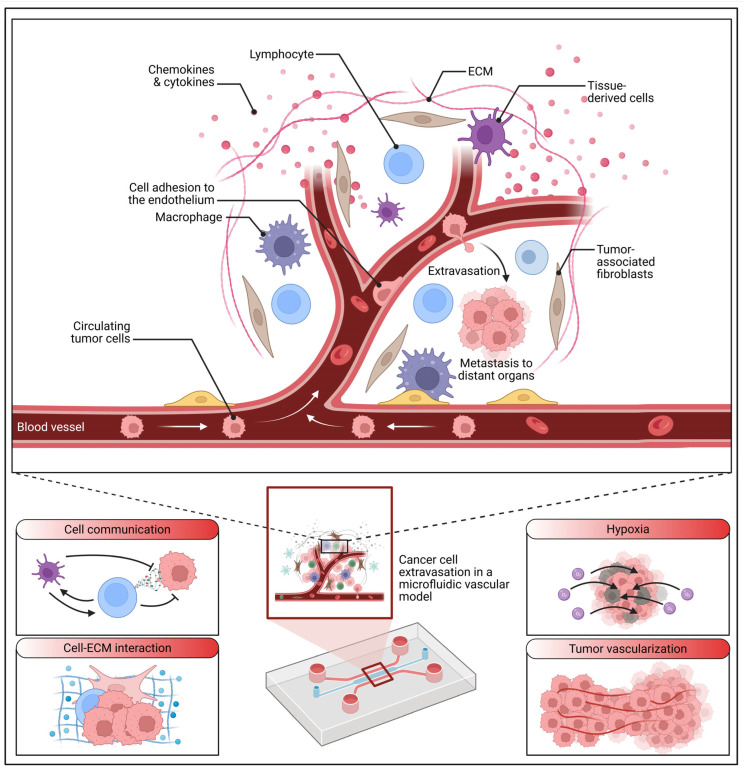
A comprehensive overview of how microfluidic devices are used to replicate the TME and simulate the complex interplay of physical and biochemical factors that influence tumor development, progression, and metastasis. The upper portion illustrates a vascularized tissue interface, where circulating tumor cells (CTCs) within blood vessels interact with endothelial cells, adhere to vessel walls, and undergo extravasation into the surrounding tissue under the influence of chemokines, cytokines, and mechanical shear forces. The TME is composed of immune cells such as macrophages and lymphocytes, tumor-associated fibroblasts, extracellular matrix (ECM), and tissue-derived stromal cells, all of which contribute to the modulation of tumor behavior and metastatic potential. These cellular and molecular components create a niche that supports cancer cell invasion and colonization at distant organs. The lower portion of the figure zooms into a microfluidic chip that mimics this vascularized environment, enabling high-resolution, in vitro modeling of cancer cell extravasation. This model captures critical TME features such as intercellular communication, cell-ECM interactions, and hypoxic gradients, which are essential for understanding tumor physiology. The chip also allows the reproduction of biochemical gradients like oxygen levels and the formation of vascular-like networks, simulating tumor angiogenesis. Overall, the microfluidic platform provides a dynamic and tunable environment to study tumor cell behavior under physiologically relevant conditions, including mechanical shear stress, hypoxia, vascularization, and stromal interactions, making it a powerful tool for cancer research, therapeutic testing, and personalized medicine development. Created by BioRender.

Emerging technologies, such as microfluidics, are increasingly contributing to the sophisticated modeling of cancer dormancy (Figure 5). Microfluidic devices afford researchers unparalleled precise control over the cellular microenvironment, thereby enabling meticulous study of the effects of various factors, including flow, shear stress, and nutrient gradients, on cancer cell behavior.

These advanced systems offer the significant potential to bridge the existing gap between traditional in vitro and in vivo models, providing a more dynamic and physiologically relevant platform for dormancy research. The utility of such systems has been demonstrated through circulating tumor cell plasticity analysis [203].

Beyond microfluidics, other advanced methodologies are also shedding light on the metastatic niche. Investigations have explored the role of specific signaling molecules and cellular interactions within this complex environment. For instance, immunotherapeutic IL-6R and targeting the MCT-1/IL-6/CXCL7/PD-L1 circuit have been found to prevent relapse and metastasis of TNBC [167].

Additional investigations have leveraged advanced techniques and model systems to further elucidate the complexities of the metastatic niche. Versatile ginsenoside Rg3 liposomes, for example, inhibit tumor metastasis by capturing circulating tumor cells and disrupting metastatic niches [175]. Osteoclast-derived apoptotic bodies inhibit naive CD8+ T cell activation via Siglec15, promoting BC secondary metastasis [176]. A microphysiological early metastatic niche-on-a-chip has revealed how heterotypic cell interactions and inhibition of integrin subunit β3 impact BC cell extravasation [177], and morphological phenotyping of organotropic brain- and bone-seeking triple-negative metastatic breast tumor cells has also been performed [178]. Furthermore, a 3D vascularized BC model has been utilized to study the role of osteoblasts in the formation of a pre-metastatic niche [179], and thyroid status has been found to regulate the TME, delineating BC fate [180]. Computed tomography has revealed microenvironment changes in the premetastatic lung [181], and micellar nanoparticles inhibit BC and pulmonary metastasis by modulating the recruitment and depletion of myeloid-derived suppressor cells [182].

### 4.4. Knowledge Gaps and Critical Analysis of Current Models

Despite significant advancements in experimental models, several critical knowledge gaps persist, hindering our comprehensive understanding of cancer dormancy and the development of effective therapies. One major limitation is the absence of in vitro models capable of faithfully recapitulating the extended periods of dormancy observed in human patients. While 3D cultures and organoids represent a substantial improvement over 2D cultures, maintaining cells in a dormant state for months or years in vitro remains a formidable challenge. Furthermore, while mouse models offer invaluable insights, they do not always accurately reflect the intricate complexities of human cancer, particularly in the context of long-term dormancy. This disparity necessitates the rigorous development of novel model systems that can better mimic the human disease course.

Another critical gap is the absence of standardized assays for defining and accurately measuring dormancy. Dormancy is recognized as a complex and heterogeneous state, yet there is currently no universally accepted set of criteria for identifying dormant cells or quantifying their behavior. This lack of standardization impedes the comparison of results across different studies and consequently hinders progress in the field. As noted, the late effects of adjuvant chemotherapy underscore the inherent complexity of dormancy in BC [204].

Current experimental models provide valuable insights into specific facets of cancer dormancy, but it is imperative to critically evaluate their inherent validity and limitations. In vitro models, while offering controlled environments, frequently lack the systemic factors and intricate interactions present in vivo. Conversely, in vivo models, while more physiologically relevant, may not fully recapitulate the entirety of human disease. The modulation of the TME drives cancer immune escape dynamics, a critical factor that in vitro models may struggle to fully replicate [205]. Consequently, the selection of a model system must be carefully considered based on the precise research question, and results should always be interpreted with caution, explicitly acknowledging the limitations of the chosen model. The imperative to integrate basic science and medical progress in BC research, a process that necessitates careful consideration of model systems, has been previously emphasized [206].

To effectively address the limitations of current models and advance our understanding of cancer dormancy, next-generation models should strive to meet several key criteria. First, they must aim to better recapitulate the human TME, encompassing the complex interactions between cancer cells, stromal cells, and the immune system. For example, anti-tumor immunity is known to rely on targeting tissue homeostasis through monocyte-driven responses [207]. Second, these models should facilitate long-term studies of dormancy, thereby allowing researchers to investigate the intricate mechanisms that govern extended periods of quiescence and subsequent recurrence. Third, the models should be amenable to high-throughput screening, enabling the systematic identification of novel therapeutic targets and the development of innovative treatment strategies. Fourth, the standardization of assays and definitions of dormancy is crucial to enhance the reproducibility and comparability of research findings across diverse studies. Finally, the development of models that incorporate patient-derived cells and tissues will significantly enhance their translational relevance. Autophagy is required for mammary tumor recurrence [86], and AMPK activation by metformin promotes the survival of dormant ER+ BC cells [208], highlighting the importance of understanding specific survival mechanisms and the mechanisms that govern the transition from dormancy to active proliferation. Local and distant tumor dormancy during early-stage BC is associated with the predominance of infiltrating T effector subsets [32], indicating that future models might benefit from incorporating immune components. Furthermore, the convergence of obesity and menopause in escape from breast tumor dormancy has been demonstrated [209], suggesting that models incorporating metabolic and hormonal factors could also be beneficial.

## 5. Clinical Translation and Biomarkers

The successful clinical translation of research findings in BC critically hinges upon the identification and rigorous validation of robust biomarkers. These biomarkers are indispensable for enhancing diagnostic precision, predicting patient prognosis, and guiding optimal treatment strategies. A significant area of intensified focus in recent years has been the comprehensive investigation of circulating tumor DNA (ctDNA) as a particularly promising biomarker.

### 5.1. Circulating Tumor DNA (ctDNA) as a Prognostic and Predictive Biomarker

The clinical utility of ctDNA in BC has been extensively explored across various studies. For instance, the prognostic and predictive value of baseline mutations and ctDNA dynamics has been demonstrably established in patients with ER-positive/HER2-negative metastatic BC [210]. Furthermore, detailed cell-free tumor DNA analysis in patients with advanced or metastatic BC has provided critical insights into mutation frequencies, testing intentions, and overall clinical impact [211]. The assessment of CSF ctDNA has notably improved diagnostic accuracy and therapeutic monitoring in BC leptomeningeal metastasis, underscoring the substantial potential of ctDNA in central nervous system involvement [212]. Insights derived from liquid biopsies have also facilitated the molecular profiling of endocrine resistance in HR+/HER2-metastatic BC, leveraging both extracellular vesicle-derived DNA and ctDNA [213]. Additionally, specific mutation patterns and ctDNA-derived prognostic markers have been rigorously identified in advanced BC patients [214].

The role of ctDNA in predicting treatment response and disease progression has also been meticulously examined. The persistent presence of ctDNA in patients with BC during neoadjuvant treatment, for example, has been identified as a significant predictor of suboptimal tumor response [215]. Genomic alterations and ctDNA dynamics have been directly linked to the early detection of progression during CDK4/6 inhibitor therapy in advanced BC [216]. Further research has focused on the mutational analysis of ctDNA in patients with estrogen receptor-positive/human epidermal growth factor receptor 2-negative advanced BC receiving palbociclib [217]. The potential for integrating personalized, ultrasensitive ctDNA monitoring to reduce imaging requirements in metastatic BC patients has been explored [218]. Methodological advancements enabling sensitive and precise detection of ctDNA through somatic copy number aberrations have also been detailed [219]. The utility of the Plasma-SeqSensei BC In vitro Diagnostics Assay for disease progression surveillance has been thoroughly discussed [220]. Furthermore, the application of ctDNA for treatment monitoring has been successfully demonstrated in a patient with synchronous metastatic angiosarcoma and BC [221].

### 5.2. Technological Advancements and Broader Biomarker Landscapes

Technological advancements have played a pivotal role in significantly enhancing the precision and scope of ctDNA analysis. Whole-genome bisulfite sequencing, for instance, has been effectively utilized for the detection and molecular classification of cancer [222]. Moreover, whole-genome circulating tumor DNA methylation landscape analysis has revealed sensitive biomarkers for BC [223]. Innovative approaches include the development of a tetrahedral DNA nanostructure-based biosensor for high-performance detection of circulating tumor DNA [224]. Discussions have also addressed the appropriate use of comprehensive cancer genome profiling assays utilizing circulating tumor DNA [225].

Beyond ctDNA, other studies have broadened the scope of the investigation to include various biomarkers and clinical scenarios. The combined assessment of circulating tumor cell (CTC) and ESR1 status in liquid biopsy samples has been highlighted for its potential to enhance the predictive clinical value in metastatic BC [226]. Serial monitoring of genomic alterations in circulating tumor cells of ER-positive/HER2-negative advanced BC has also been explored [227]. Comparative analyses between circulating tumor DNA and extracellular vesicle DNA have further expanded our understanding of liquid biopsy components [228]. Methodologies for modeling clonal structure over narrow time frames via circulating tumor DNA have contributed to resolving tumor evolution dynamics [229]. Undetectable circulating tumor DNA levels have been found to correlate with a low risk of recurrence/metastasis in postoperative pathologic stage I lung adenocarcinoma patients [230]. A comprehensive cell-free DNA comparative analysis has also been conducted [231]. Clinicopathologic features, genomic profiles, and outcomes have been thoroughly examined concerning these biomarkers [123]. Furthermore, PIK3CA mutations have been a specific focus of some research endeavors [232].

### 5.3. Biomarkers in Guiding Treatment Strategies

The integration of biomarker analysis into treatment strategies represents a critical step towards personalized medicine. Several studies have investigated therapeutic approaches in the context of specific biomarker profiles. For example, ATR inhibitor camonsertib dose optimization in biomarker-selected advanced solid tumors has been thoroughly explored [233]. A phase II study investigating the MEK inhibitor trametinib in combination with the AKT inhibitor uprosertib has been conducted in metastatic TNBC [234]. Additionally, a phase II study of anlotinib in pre-treated HER-2 negative metastatic BC has been presented [235]. A case study documenting the activity of atezolizumab has also been reported [236]. These collective studies underscore the increasing importance of ctDNA and other biomarkers in the clinical management of BC, actively driving the field towards more personalized and ultimately more effective treatment approaches (Table 1).

### 5.4. Patient-Specific Variables Influencing Breast Cancer Dormancy

While the inherent heterogeneity of breast cancer disseminated tumor cells (DTCs) is widely acknowledged, a truly comprehensive understanding of dormancy—the prolonged quiescent state preceding metastatic relapse—demands a deeper consideration of how patient-specific variables intricately sculpt this process. Beyond the intrinsic characteristics of the tumor cells themselves, factors such as a patient’s age, menopausal status, and underlying genetic background fundamentally alter the systemic endocrine and immune environments, thereby profoundly impacting DTC behavior and the critical support provided by the host microenvironment for dormancy maintenance or its eventual disruption.

The systemic endocrine environment, profoundly influenced by a patient’s age and menopausal status, plays a crucial, yet often underappreciated, role in dictating the fate of DTCs. For instance, the fluctuating hormonal milieu associated with pre- or post-menopausal states can directly modulate signaling pathways within DTCs, influencing their proliferative capacity, stress resilience, and propensity for quiescence. These hormonal shifts also contribute to dynamic changes within the tumor microenvironment (TME) itself, altering the composition and activity of immune cells and stromal components in a manner that can either promote or hinder dormancy. While direct causal links between specific menopausal changes and dormancy mechanisms are still an active area of investigation, it is plausible that age-related shifts in systemic inflammation or immune senescence could create distinct “niches” that either favor or challenge DTC survival and dormancy. Indeed, the broader understanding of how the TME modulates cancer immune escape dynamics [1] underscores the potential for systemic host factors to influence dormancy by reshaping these intricate local interactions.

Beyond systemic influences, the genetic background of the host, encompassing both germline and somatic alterations, exerts a profound impact on dormancy programs. Germline genetic variations can predispose individuals not only to specific tumor types but also to distinct host immune responses or TME compositions that may inherently favor or counteract dormancy. Such predispositions could influence the initial establishment of a dormant state or the likelihood of reactivation. More overtly, somatic genetic alterations within the tumor cells themselves critically influence their intrinsic dormancy programs. For example, the elegant observation that long-term breast cancer response to CDK4/6 inhibition is profoundly defined by TP53-mediated geroconversion [238] highlights how a specific somatic mutation can dictate a dormancy-like, senescent state. This suggests that certain genomic aberrations within DTCs might intrinsically favor a quiescent phenotype, making them less susceptible to conventional therapies. Conversely, the loss of specific genes, such as Brd7, has been shown to paradoxically “reawaken” dormant metastasis-initiating cells in the lung by forging an immunosuppressive niche [174].This vividly illustrates how specific genetic events can disrupt dormancy maintenance and trigger metastatic outgrowth.

Furthermore, these genetic characteristics intricately interact with the host microenvironment to shape the dormancy landscape. Genomic characteristics, including those influencing histology-based immune features in breast cancer [239], suggest that the inherent genomic makeup of the primary tumor can predispose the metastatic niche to a particular immune profile, thus influencing DTC dormancy. The precise interactions between DTCs and key immune populations, such as Natural Killer (NK) cells, are critical, with NK cell regulation of breast cancer stem cells demonstrably mediating metastatic dormancy [5]. This underscores that patient-specific immune cell activity can serve as a vital host factor determining dormancy success. The local TME also adapts to specific genetic cues. For example, matrix stiffness, a microenvironmental factor, can induce a stemness-dormancy state transition in breast cancer cells [29], hinting that patient-specific variations in tissue rigidity or ECM composition could influence dormancy pathways. The initiation of tumor dormancy by the lymphovascular embolus itself [240] suggests that the very initial dissemination process, which varies between patients, can impact the subsequent dormancy potential.

Ultimately, the collective influence of these patient-specific variables creates a spectrum of “dormancy phenotypes”. The systemic environment, influenced by age and hormonal status, sets the broad context, while the precise germline and somatic genetic landscapes dictate the intrinsic dormancy programs of DTCs and their nuanced interactions with organ-specific microenvironments. For instance, lung-resident alveolar macrophages are now understood to critically regulate the timing of breast cancer metastasis [241], emphasizing how organ-specific immune cells within the host can be decisive. Similarly, tryptophan 2,3-dioxygenase (IDO)-positive matrix fibroblasts have been implicated in fueling breast cancer lung metastasis by fostering kynurenine-mediated ferroptosis resistance in metastatic cells and inducing T cell dysfunction [133], illustrating a complex, patient-context-dependent TME interaction. These intricate cell-cell interactions mediating primary and metastatic breast cancer dormancy are complex and highly individualized [242]. This profound heterogeneity underscores why integrating the biology of breast cancer cell dormancy into clinical practice is a significant challenge [13] yet also represents a crucial step towards personalized strategies for preventing metastatic relapse [243].

The challenges inherent in monitoring this patient-specific dormancy are also significant. While circulating tumor DNA (ctDNA) holds immense promise for early relapse detection [244] and can even be used for monitoring treatment response in preclinical models, its utility for precisely distinguishing dormant from activated states remains a critical limitation [245]. The low turnover of quiescent dormant cells often results in minimal or undetectable ctDNA shedding, making it challenging to track dormancy status. However, advanced approaches like assessing differential methylation of circulating free DNA are emerging as tools for breast cancer diagnosis and even BRCA1/2 mutation detection [2], hinting at future possibilities for dormancy-specific liquid biopsies that might overcome current ctDNA limitations. This understanding of patient-specific influences is pivotal for developing truly personalized surveillance and intervention strategies.

## 6. Future Directions and Research Priorities

The persistent challenge of late metastatic recurrence in BC, driven by dormant DTCs, unequivocally underscores the urgent need for innovative research to unravel dormancy mechanisms and translate these critical findings into tangible clinical practice [246]. While current knowledge highlights the profound roles of molecular pathways, intricate microenvironmental cues, and dynamic immune interactions in mediating dormancy, significant gaps, such as the elusive nature of reactivation triggers, the limitations of existing models, and the scarcity of dedicated clinical trials, continue to impede substantial progress [247]. This section outlines future research priorities, meticulously designed to address these prevailing gaps. These priorities strategically focus on several key areas: deepening molecular understanding through advanced omics approaches, dissecting microenvironmental dynamics, developing more sophisticated experimental models, accelerating clinical translation, and fostering interdisciplinary collaborations. By seamlessly integrating cutting-edge technologies and adopting truly interdisciplinary strategies, these priorities aim to bridge the often-cited chasm between preclinical discovery and clinical implementation, with the ultimate objective of significantly reducing the pervasive burden of metastatic relapse in BC.

### 6.1. Unraveling Molecular Mechanisms of Dormancy

Single-cell multi-omics, which integrates genomics, transcriptomics, epigenomics, and proteomics at the individual cell level, offers an exceptionally powerful approach to precisely mapping the molecular transitions between dormancy and subsequent reactivation [248]. Recent studies utilizing single-cell RNA sequencing (scRNA-seq) have already begun to identify distinct DTC subpopulations exhibiting characteristic quiescence signatures (e.g., elevated p38 MAPK and reduced ERK MAPK activity) in ER+ BC [249,250]. However, the robust integration of these transcriptional findings with complementary epigenetic and proteomic data remains in its nascent stages. While multi-omic profiling of bone marrow DTCs has already revealed subtype-specific heterogeneity, with TNBC DTCs notably demonstrating Wnt pathway activation [251], longitudinal studies capable of tracking these intricate transitions in vivo are conspicuously lacking. Future research endeavors should prioritize the development of integrated platforms, such as single cell CUT&Tag for detailed epigenomic profiling, to meticulously map H3K27me3 dynamics during the process of reactivation. Such advanced approaches hold the potential to identify novel therapeutic targets, such as specific transcription factors (e.g., NR2F1), that are critically involved in driving the exit from dormancy.

Non-coding RNAs (ncRNAs), encompassing both microRNAs (miRNAs) and long non-coding RNAs (lncRNAs), are increasingly recognized as pivotal regulators of dormancy, yet their precise roles remain significantly underexplored. For example, while miR-223 has been shown to suppress reactivation in TNBC by targeting EMT pathways, its specificity for ER+ BC remains unclear [252]. Conversely, lncRNA MALAT1 has been implicated in modulating chromatin accessibility in dormant ER+ DTCs, suggesting its compelling therapeutic potential [253]. Future investigations should strategically employ CRISPR-based screens to systematically identify these complex ncRNA networks and their intricate epigenetic interactions, leveraging powerful tools such as single-cell ATAC-seq to precisely map chromatin states. Furthermore, epigenetic therapies, including EZH2 inhibitors, have demonstrated significant preclinical promise in maintaining dormancy, thus necessitating urgent clinical validation [254]. These concerted efforts promise to uncover subtype-specific epigenetic signatures, paving the way for highly targeted and effective interventions.

### 6.2. Dissecting Microenvironmental Control of Dormancy

Spatial transcriptomics, a revolutionary technology that maps gene expression within its native tissue context, is fundamentally transforming our comprehension of metastatic niche dynamics. Recent studies employing Visium spatial transcriptomics have compellingly revealed macrophage polarization gradients within bone marrow niches, with M2 macrophages predominantly associated with ER+ dormancy [255]. However, comprehensive temporal analyses of niche remodeling, such as dynamic shifts in ECM composition or immune cell infiltration, are currently limited [256]. Future research should prioritize integrating spatial transcriptomics with rigorous time-course studies to precisely track M1/M2 macrophage transitions and the contributions of crucial stromal cells (e.g., cancer-associated fibroblasts, CAFs) across diverse metastatic sites, including bone marrow and lung. Combining spatial gene expression data with proteomic analyses could further elucidate critical cytokine gradients (e.g., IL-10, TGF-β) that actively drive dormancy, thereby informing the development of targeted niche-modulating therapies [257]. Understanding the intricate interactions between immune cells and the broader TME is undeniably crucial for developing effective strategies to modulate dormancy and ultimately prevent metastatic relapse in BC. By precisely focusing on these dynamic interplays, researchers can identify potent therapeutic targets that promise to enhance treatment efficacy for BC patients.

Macrophage-targeted therapies, such as CSF1R inhibitors, hold significant potential to modulate dormancy by selectively altering M2 macrophage polarization, although their efficacy appears context-dependent. Preclinical trials indicate that CSF1R inhibition prolongs ER+ dormancy by reducing immunosuppressive M2 macrophages. Intriguingly, however, it can accelerate TNBC reactivation through M1-driven inflammation [258]. Future studies should rigorously explore combination therapies, perhaps pairing CSF1R inhibitors with anti-PD-L1 antibodies to strategically enhance T-cell surveillance. Furthermore, the judicious application of nanoparticle-based delivery systems for macrophage-reprogramming agents (e.g., IL-4 inhibitors) could significantly improve therapeutic specificity, thereby warranting early-phase clinical trials [259]. Critically, investigating macrophage-T cell crosstalk using sophisticated co-culture models will be instrumental in clarifying their precise roles in niche remodeling and overall immune modulation [260]. A deeper understanding of these cellular interactions is essential for developing innovative therapeutic strategies that can effectively target the dormant state of DTCs and prevent the consequences of metastatic recurrence.

### 6.3. Advancing Experimental Models for Dormancy Research

Current experimental models largely fail to recapitulate the decades-long latency observed in human ER+ BC, thereby necessitating the development of more accurate humanized, long-term systems. Organ-on-chip platforms, meticulously designed to mimic specific bone marrow or lung niches, have demonstrated the ability to induce dormancy via factors like hypoxia and TGF-β, however, these cultures are typically sustainable for only weeks [261]. Future research must prioritize the development of advanced bioreactors or cryopreservation-compatible organoids to extend the duration of dormancy induction to months or even years. Crucially, these models should incorporate human immune cells (e.g., NK cells, macrophages) to ensure accurate immune competence. Humanized mouse models, engrafted with patient-derived tumors and human immune cells, show considerable promise for studying complex phenomena like PD-L1-mediated immune evasion, though scalability remains a significant challenge [262]. Ultimately, these more refined models could serve as invaluable platforms for validating novel therapies, such as integrin inhibitors, over clinically relevant timelines. Developing innovative long-term models is crucial for studying the complex and dynamic interactions within the TME and their profound impact on dormancy and subsequent reactivation in BC.

Standardizing Dormancy Assays: The current absence of standardized dormancy assays severely hampers the reproducibility and comparability of research findings across different laboratories. Existing assays, often relying on simplistic readouts like Ki67 negativity or p27 expression, frequently lack the necessary specificity to reliably predict reactivation risk [263]. Future research must focus on developing multi-parametric assays that integrate diverse readouts, including molecular markers (e.g., p38/ERK ratio), functional assessments (e.g., time-lapse microscopy for reactivation kinetics), and advanced imaging-based techniques (e.g., FRET sensors). Furthermore, high-throughput platforms, such as microfluidic systems, could play a pivotal role in standardizing quiescence induction across various cell lines (ER+, TNBC) and distinct metastatic niches [264]. Establishing universally accepted and rigorously standardized dormancy assays will significantly enhance the reliability and interpretability of research findings, ultimately accelerating the development of truly effective therapies to combat metastatic recurrence in BC.

### 6.4. Accelerating Clinical Translation

Clinical trials specifically targeting dormant cells are critically needed but remain notably underdeveloped. Strategies focused on dormancy maintenance, such as integrin β1 agonists, have demonstrated compelling preclinical promise by sustaining quiescence, and Phase I trials are currently exploring their safety profiles [265]. Conversely, approaches aimed at DTC eradication, such as autophagy inhibitors (e.g., hydroxychloroquine), target quiescent cells but frequently encounter significant toxicity challenges [266]. Combination immunotherapies, pairing anti-PD-1 agents with CSF1R inhibitors, could potentially enhance DTC clearance by boosting T cell activity, thus warranting pilot studies [267]. Crucially, future trial designs must incorporate dormancy-specific endpoints, such as time-to-reactivation, and stratify patients by BC subtype (e.g., ER+ versus TNBC) to effectively address inherent heterogeneity and optimize therapeutic strategies [268]. By focusing on these distinct clinical challenges, future trials can more effectively evaluate the efficacy of targeted therapies against dormant DTCs in BC, thereby addressing the complexities of dormancy and significantly improving patient outcomes. Ultimately, innovative trial designs and robust interdisciplinary collaborations are essential for translating fundamental research into impactful clinical interventions.

The rigorous validation of biomarkers for predicting reactivation risk is paramount but necessitates well-designed longitudinal patient cohorts. While ctDNA profiling is adept at detecting minimal residual disease (MRD), it often lacks the requisite specificity for precisely discerning dormancy transitions. In contrast, CTC transcriptomics has identified NR2F1 as a promising candidate marker, though this requires further validation in prospective studies [269]. Future research should prioritize the establishment of large, multi-center cohorts that meticulously track ctDNA/CTC changes over extended periods (e.g., 5–20 years), leveraging machine learning algorithms to seamlessly integrate multi-omic data (e.g., mutations, methylation patterns). Epigenetic biomarkers, such as H3K27me3, should be rigorously validated in prospective studies, utilizing highly sensitive techniques like ddPCR for enhanced detection [270,271]. These comprehensive efforts are poised to enable more accurate risk stratification and the implementation of truly personalized monitoring strategies for patients at risk of metastatic recurrence.

### 6.5. Fostering Interdisciplinary Approaches

Computational modeling, particularly agent-based models (ABMs), offers a powerful and predictive tool for simulating dormancy dynamics by explicitly modeling DTC-niche interactions. Recent ABMs have successfully modeled macrophage polarization and T-cell surveillance, predicting reactivation thresholds within ER+ niches [272]. However, the robust integration of these computational models with experimental data remains limited, and current models often oversimplify the inherent heterogeneity of stromal components [273]. Future research should prioritize coupling ABMs with high-resolution spatial transcriptomics and scRNA-seq data to realistically simulate temporal niche remodeling, with validation against patient-derived organoids. Machine learning techniques could further refine these models by identifying critical drivers (e.g., TGF-β gradients), thereby providing invaluable guidance for subsequent experimental design [274]. These interdisciplinary approaches hold immense promise for accelerating therapeutic discovery and optimizing crucial trial endpoints. By prioritizing these innovative research directions, the scientific community can significantly enhance its understanding and treatment of dormancy in BC, ultimately aiming to improve patient survival and overall quality of life.

Future research in BC dormancy must strategically leverage single-cell multi-omics and comprehensive ncRNA/epigenetic studies to meticulously map molecular transitions. Spatial transcriptomics and macrophage-targeted therapies are essential for dissecting niche dynamics. Furthermore, the development of humanized, long-term models and the standardization of dormancy assays are paramount. Concurrently, dormancy-focused clinical trials must be meticulously designed, and computational modeling should be seamlessly integrated with experimental data to maximize discovery. These priorities, firmly grounded in interdisciplinary innovation, are designed to comprehensively address critical gaps in dormancy research, thereby paving the way for the discovery of novel biomarkers and the development of breakthrough therapies to prevent metastatic relapse and profoundly improve patient outcomes.

### 6.6. Bridging the Translational Gap

The effective clinical management of breast cancer dormancy hinges critically on the ability to accurately monitor and predict the behavior of disseminated tumor cells (DTCs). In this context, circulating tumor DNA (ctDNA), a promising component of liquid biopsies, has garnered significant attention for its non-invasive nature and utility in cancer management [275,276,277,278,279]. It provides a powerful tool for detecting tumor presence, monitoring treatment response, and tracking cancer evolution throughout the disease course [280]. However, applying ctDNA specifically to dormancy presents inherent limitations. While valuable in actively proliferating disease, ctDNA often struggles to distinguish between quiescent, non-proliferating dormant cells and actively proliferating, activated tumor cells [245]. This inability stems from the minimal proliferative activity of dormant cells, which leads to very low or even undetectable ctDNA shedding [245]. Consequently, relying solely on ctDNA for monitoring dormancy status, predicting reactivation events, or guiding dormancy-specific treatment decisions remains a significant challenge in breast cancer management [281,282]. Overcoming this requires novel approaches capable of identifying the unique molecular signatures of quiescent cells.

To address these complex challenges and accelerate the translation of dormancy research, computational modeling offers innovative and powerful application scenarios within future directions. Beyond general data integration, machine learning and artificial intelligence (AI) can be leveraged for advanced predictive modeling. These approaches can analyze complex, multi-omics datasets (e.g., genomics, transcriptomics, proteomics) derived from dormant cells and their microenvironment to predict the likelihood of dormancy, estimate the time to recurrence, or forecast responsiveness to novel dormancy-specific interventions. Furthermore, simulation of tumor microenvironment (TME) dynamics using agent-based or mechanistic models can elucidate the intricate, often non-linear interactions between dormant cells, various immune components, and stromal elements. This capability allows researchers to identify critical tipping points that govern dormancy maintenance or trigger reactivation, offering insights unattainable through purely experimental methods. Computational approaches are also instrumental in the identification of novel pathways and drug targets. Through sophisticated network analysis and systems biology methodologies, these models can integrate diverse datasets to uncover previously unknown dormancy pathways or therapeutic targets that might sensitize quiescent cells. Finally, computational modeling is poised to revolutionize therapeutic optimization. By simulating the effects of different agents, including TME-targeting agents or autophagy inhibitors, in combination with existing treatments, these models can predict synergistic effects and optimize combination therapy regimens for maximum efficacy against dormant cell populations. These innovative applications of computational modeling are crucial for developing a more nuanced understanding of dormancy, overcoming current biomarker limitations, and designing highly effective, personalized strategies to prevent metastatic relapse.

## 7. Conclusions

The formidable challenge of late metastatic recurrence in BC, especially in ER+ subtypes where relapses can emerge decades after initial treatment, is profoundly driven by dormant DTCs. This review emphatically highlights the urgent need to unravel tumor dormancy, a critical factor contributing significantly to global BC mortality. Dormant DTCs, residing within protective niches like the bone marrow or circulating through the bloodstream, deftly evade conventional therapies and immune surveillance. Their unpredictable reactivation underscores the pressing need for targeted strategies to either permanently maintain their quiescence or, ideally, eradicate them. The devastating impact of these late recurrences, affecting a substantial portion of ER+ patients, compels immediate and focused research to fully dissect the molecular, microenvironmental, and clinical complexities of dormancy.

Current dormancy research remains somewhat fragmented, plagued by conflicting data on immune modulation, inadequate long-term experimental models, and a notable scarcity of reliable predictive biomarkers. This necessitates a decisive shift towards integrated and standardized approaches. While molecular studies have begun to reveal the intricate roles of p38/ERK signaling, epigenetic reprogramming, and non-coding RNAs, the precise triggers for dormancy reactivation remain elusive. Similarly, microenvironmental investigations have illuminated dynamic cellular interactions, yet a comprehensive understanding of temporal niche dynamics and stromal heterogeneity across different metastatic sites remains poorly defined. Existing experimental models consistently fall short of capturing the prolonged latency of human dormancy, and the absence of standardized assays undeniably hinders reproducibility. Clinically, the scarcity of dedicated trials and the limitations of current biomarker profiling for precise dormancy monitoring signify critical translational gaps. Addressing these challenges demands a cohesive research framework integrating advanced multi-omics, spatial profiling, and computational modeling to comprehensively map dormancy transitions, alongside the establishment of standardized assays for cross-study comparability.

Ultimately, tackling BC dormancy requires a paradigm shift towards integrated, standardized, and patient-centric research. By aligning molecular insights with microenvironmental understanding and clinical efforts, the field can develop novel biomarkers to reliably stratify reactivation risk and devise groundbreaking therapies to definitively prevent metastatic relapse. This review serves as a powerful catalyst, guiding researchers, clinicians, and policymakers to prioritize dormancy as a preeminent frontier in BC, with the profound potential to dramatically improve outcomes and enhance the quality of life for millions of patients worldwide.

## Figures and Tables

**Table 1:** Clinical Utility and Technological Advancements of Biomarkers in BC.

Biomarker Type	Clinical Application/Focus	Specific Findings/Methodology	Citation
**ctDNA**	Prognostic and Predictive Value (General)	Baseline mutations and ctDNA dynamics as prognostic/predictive factors in ER+/HER2- metastatic BC.	[210]
**ctDNA**	Mutation Frequencies, Testing Intentions, Clinical Impact	Cell-free tumor DNA analysis in advanced/metastatic BC patients.	[211]
**ctDNA (CSF)**	Diagnostic and Therapeutic Monitoring	Improved diagnostic accuracy and therapeutic monitoring in BC leptomeningeal metastasis.	[212]
**ctDNA (EV-derived)**	Endocrine Resistance Profiling	Molecular profiling of endocrine resistance in HR+/HER2- metastatic BC using EV-derived DNA and ctDNA.	[213]
**ctDNA**	Prognostic Markers	Identification of mutation patterns and ctDNA-derived prognostic markers in advanced BC patients.	[214]
**ctDNA**	Predicting Treatment Response (Neoadjuvant)	The persistence of ctDNA during neoadjuvant treatment predicts poor tumor response.	[215]
**ctDNA**	Early Detection of Progression (CDK4/6 Inhibitors)	Genomic alterations and ctDNA dynamics linked to early detection of progression on CDK4/6 inhibitor therapy.	[216]
**ctDNA**	Mutational Analysis (Palbociclib)	Mutational analysis in ER+/HER2- advanced BC patients receiving palbociclib.	[217]
**ctDNA**	Reducing Imaging Requirements	Integration of personalized ultrasensitive ctDNA monitoring to potentially reduce imaging needs in metastatic BC.	[218]
**ctDNA**	Sensitive Detection Method	Methodological advancements for sensitive and precise ctDNA detection through somatic copy number aberrations.	[219]
**ctDNA**	Disease Progression Surveillance Assay	Discussion of Plasma-SeqSensei BC In vitro Diagnostics Assay for surveillance of disease progression.	[220]
**ctDNA**	Treatment Monitoring (Synchronous Cancers)	Treatment monitoring using ctDNA in a patient with synchronous metastatic angiosarcoma and BC.	[221]
**ctDNA**	Sensitive Biomarkers	Whole-genome circulating tumor DNA methylation landscape analysis revealing sensitive biomarkers.	[223]
**ctDNA**	High-Performance Detection	Development of a tetrahedral DNA nanostructure-based biosensor for high-performance ctDNA detection.	[224]
**Circulating Tumor Cells (CTCs)**	Serial Monitoring of Genomic Alterations	Serial monitoring of genomic alterations in circulating tumor cells of ER+/HER2- advanced BC.	[227]
**ctDNA vs. Extracellular Vesicle DNA**	Comparative Analysis	Comparative analyses between ctDNA and extracellular vesicle DNA.	[228]
**ctDNA**	Modeling Clonal Structure	Modeling clonal structure over narrow time frames via circulating tumor DNA.	[229]
**Undetectable ctDNA**	Recurrence/Metastasis Risk	Undetectable ctDNA levels correlate with a low risk of recurrence/metastasis in postoperative stage I lung adenocarcinoma.	[230]
**Cell-free DNA (cfDNA)**	Comparative Analysis	Comprehensive cell-free DNA comparative analysis.	[231]
**Multi-omics**	Clinicopathologic Features, Genomic Profiles, Outcomes	Examination of clinicopathologic features, genomic profiles, and outcomes concerning biomarkers.	[237]
***PIK3CA*** **Mutations**	Specific Mutation Focus	Research focusing on *PIK3CA* mutations.	[232]
**Biomarkers in Guiding Treatment**	ATR Inhibitor (Camonsertib)	Dose optimization in biomarker-selected advanced solid tumors.	[233]
**MEK/AKT Inhibitors (Trametinib/Uprosertib)**	Phase II Study (mTNBC)	Phase II study in metastatic TNBC.	[234]

## Data Availability

Not applicable.

## Abbreviations

**ABMs**: agent-based models; **CDK**: cyclin-dependent kinases; **CDKI**: cyclin-dependent kinase inhibitors; **CTC**: circulating tumor cells; **ddPCR**: droplet digital PCR; **DTC**: disseminated tumor cell; **ECM**: ECM; **ER+**: estrogen receptor-positive; **FAO**: fatty acid oxidation; **FRET**: Förster resonance energy transfer; **HDAC**: histone deacetylase; **lncRNA**: long non-coding RNA; **MAPK**: mitogen-activated protein kinase; **MDSC**: myeloid-derived suppressor cell; **miRNA**: microRNA; **MRD**: minimal residual disease; **MSC**: mesenchymal stem cell; **ncRNA**: non-coding RNA; **NET**: neutrophil extracellular trap; **NK cell**: natural killer cell; **OXPHOS**: oxidative phosphorylation; **scRNA-seq**: single-cell RNA sequencing; **TAM**: tumor-associated macrophage; **TGF-β**: transforming growth factor-beta; **TME**: tumor microenvironment; **TNBC**: triple-negative breast cancer; **CTDNA**: circulating tumor DNA; **PD-L1**: programmed death-ligand 1; **EMT**: epithelial-mesenchymal transition; **CAF**: cancer-associated fibroblast; **EV**: extracellular vesicle; **LIFR**: leukemia inhibitory factor receptor; **CSF1R**: colony-stimulating factor 1 receptor; **CRISPR**: clustered regularly interspaced short palindromic repeats; **IL-4**: interleukin-4; **STAT6**: signal transducer and activator of transcription 6; **IFN-γ**: interferon-gamma; **NR2F1**: nuclear receptor subfamily 2 group F member 1; **CDH11**: cadherin-11; **ITGA5**: integrin alpha-5; **EZH2**: enhancer of zeste homolog 2; **PD-1**: programmed cell death protein 1; **HER2**: human epidermal growth factor receptor 2; **AMPK**: AMP-activated protein kinase; **GPR78**: glucose-regulated protein 78; **MCT-1**: monocarboxylate transporter 1; **IL-6R**: interleukin-6 receptor; **CXCL**: C-X-C motif chemokine ligand.
